# Enhancing Butanol Production under the Stress Environments of Co-Culturing *Clostridium acetobutylicum/Saccharomyces cerevisiae* Integrated with Exogenous Butyrate Addition

**DOI:** 10.1371/journal.pone.0141160

**Published:** 2015-10-21

**Authors:** Hongzhen Luo, Laibing Ge, Jingshu Zhang, Yanli Zhao, Jian Ding, Zhigang Li, Zhenni He, Rui Chen, Zhongping Shi

**Affiliations:** 1 The Key Laboratory of Industrial Biotechnology, Ministry of Education, School of Biotechnology, Jiangnan University, Wuxi, Jiangsu, China; 2 China Shijiazhuang Pharmaceutical Group Co., Ltd., Shijiazhuang, Hebei, China; 3 Hebei Changshan Biochemical Pharmaceutical Co., Ltd., Shijiazhuang, Hebei, China; National Taiwan University, TAIWAN

## Abstract

In this study, an efficient acetone-butanol-ethanol (ABE) fermentation strategy integrating *Clostridium acetobutylicum*/*Saccharomyces cerevisiae* co-culturing system with exogenous butyrate addition, was proposed and experimentally conducted. In solventogenic phase, by adding 0.2 g-DCW/L-broth viable *S*. *cerevisiae* cells and 4.0 g/L-broth concentrated butyrate solution into *C*. *acetobutylicum* culture broth, final butanol concentration and butanol/acetone ratio in a 7 L anaerobic fermentor reached the highest levels of 15.74 g/L and 2.83 respectively, with the increments of 35% and 43% as compared with those of control. Theoretical and experimental analysis revealed that, the proposed strategy could, 1) extensively induce secretion of amino acids particularly lysine, which are favorable for both *C*. *acetobutylicum* survival and butanol synthesis under high butanol concentration environment; 2) enhance the utilization ability of *C*. *acetobutylicum* on glucose and over-produce intracellular NADH for butanol synthesis in *C*. *acetobutylicum* metabolism simultaneously; 3) direct most of extra consumed glucose into butanol synthesis route. The synergetic actions of effective amino acids assimilation, high rates of substrate consumption and NADH regeneration yielded highest butanol concentration and butanol ratio in *C*. *acetobutylicum* under this stress environment. The proposed method supplies an alternative way to improve ABE fermentation performance by traditional fermentation technology.

## Introduction

Bio-butanol is not only an important platform chemical [1, 2], but also a clean/renewable liquid fuel [2] and powerful fuel additive [3]. Butanol fermentation is also referred as acetone-butanol-ethanol (ABE) fermentation. Nowadays, *C*. *acetobutylicum* and corn starch are still the dominated strain and substrate for industrial bio-butanol production in the world. In ABE fermentation, butanol, acetone and ethanol are roughly produced at a mole ratio of 6:3:1 (B:A:E), increasing butanol concentration and ratio without sacrificing the total solvent productivity has been the two major targets pursued by many researchers. ABE fermentation is characterized with severe butanol inhibition. To alleviate the problem, a couple of methods including strain mutagenesis, genetic engineering and metabolic regulation have been implemented, but the entire fermentation performance improvement could not reach the expected level [4]. ABE fermentation with in-situ butanol separation could significantly enhance the productivity of total solvents, particularly that of butanol [5]. However, the economics and the operation complexity of the in-situ butanol separation system limit its practical application. So far, traditional batch ABE fermentation remains the dominated operation mode for industrial bio-butanol production.

Traditional solvent products recovery or purification process costs a huge amount of energy, which limits development of ABE fermentation industry in the future. Actually, the solvents-mixture obtained in ABE fermentation could be recognized and directly used as an excellent diesel additive for ordinary diesel engine, as it could reduce natural luminosity intensity or soot formation [3]. In that report, it was showed that the fuel additive with higher butanol ratio could improve engine ignition performance or grade/quality of the diesel, when adding 20% (v/v) ABE solvents-mixture (ABE20) into the diesel. Thus, it is attractive to distill solvents-mixture with higher butanol ratio from ABE fermentation broth in an energy-saving way. Report [6] indicated that 82% of the total solvents could be extracted in a form of ABE solvents-mixture (recovery ratio: butanol 96%, acetone 64%, ethanol 50%) in an unit using 2-ethyl-1-hexanol (EH) as the extractant (volumetric ratio of EH/ABE fermentation broth, 1:1), when the solvents are produced in the well-recognized ratio (B:A:E = 6:3:1). When the extractant containing ABE was directed into a subsequent stripper unit, an acetone-butanol-ethanol ternary azeotropic system could be formed and the solvents-mixture could be 100% stripped on the top of the tower, while the expensive EH could be fully recovered at the tower bottom, allowing its repeated utilization. Production of ABE solvents-mixture based diesel additive by this kind of recovery method would make ABE fermentation process more economic and versatile in applications.

Selectively increasing butanol concentration and ratio are very important in ABE fermentation, which would have two advantages for the diesel additive bio-production: 1) improving quality of the diesel blended with ABE solvents [3]; 2) further increasing solvents extraction yield as the butanol recovery ratio is the highest (96%) with the purification system described by the literature [6]. Many research works have been conducted attempting to obtain higher butanol concentration or ratio (or butanol/acetone ratio), including utilization of mixed substrates [7], screening of hyper-butanol strains [4, 8], control of oxidative-reductive potential [9], and addition of electron carriers/pigments such as neutral red and methyl viologen [10–12] to create NADH enriched environment to enhance butanol production. However, in those cases, higher butanol ratio was obtained at the expense of reducing total ABE solvents productivity, increasing purification loads (pigments removal), etc. In ABE fermentation, *Clostridia* have the ability to simultaneously utilize glucose and butyrate to synthesize butanol. It has been well-recognized that, utilizing butyrate as the co-substrate could increase butanol concentration [13] and conversion yield of butanol/substrates [14]. In our previous studies, we enhanced butanol concentration and butanol/acetone ratio in ABE fermentations to the extents of 5∼17% and 16∼23% respectively, when using reductive power enriched cassava medium to replace corn-based medium [15] or consecutively feeding a small amount of butyrate in corn-based ABE fermentation [16]. However, the increments were limited and far below the expectation.

As an important fermentation technique, the method of co-culturing two or more than two microorganisms has been applied in a couple of bioprocesses including food manufactures, bio-degradations, and bio-fuel productions [17, 18]. The technique was also reported in a couple of studies related with ABE fermentations. A co-culturing system of *C*. *butylicum* and amylase producing *B*. *subtilis* was proposed and the system could self-generate amylase and consume dissolved oxygen, allowing ABE fermentation to be more cost effective [19]. The other report showed that, co-culturing *C*. *beijerinckii* and *C*. *tyrobutyricum* could increase butanol concentration and substrate conversion yield, as the butyrate produced by *C*. *tyrobutyricum* could be effectively utilized by *C*. *beijerinckii* as a more efficient substrate [20]. Among the potential co-culture systems for ABE fermentation, adaptively adding viable *S*. *cerevisiae* cells into *C*. *acetobutylicum* culture broth could be considered as a special co-culturing system and promising protocol for enhancing ABE fermentation performance. The translation process of *S*. *cerevisiae* was seriously restricted under high butanol concentration environment and the amino acids pools were preserved [21]. There is a possibility to improve ABE fermentation performance by co-culturing *C*. *acetobutylicum* with *S*. *cerevisiae*: 1) the amino acids might be secreted by *S*. *cerevisiae* under its stress environment and then assimilated by *C*. *acetobutylicum*, and some of them may be favorable for *C*. *acetobutylicum* survival and butanol synthesis; 2) for cells survival, *C*. *acetobutylicum* has to compete with the yeast on substrate utilization, so that substrate consumption amount by *C*. *acetobutylicum* would be enhanced and more intracellular NADH production required for butanol synthesis could be expected.

In this study, attempting to further enhance butanol concentration and ratio in corn-based ABE fermentation, we proposed a novel fermentation strategy of co-culturing *C*. *acetobutylicum*/*S*. *cerevisiae* integrated with exogenous butyrate addition, which incorporated the advantages of the co-culturing system and butyrate addition. The effectiveness of the proposed strategy was testified in both anaerobic bottles and fermentor.

## Materials and Methods

### Microorganisms

*Clostridium acetobutylicum* ATCC 824 was maintained in the form of spore suspension in 5% (w/v) corn meal medium at 4°C. The methods of inoculation and pre-culture were the same as those previously described [22]. *Saccharomyces cerevisiae* was obtained from Angel Yeast Co., China. It was maintained at 4°C on Yeast extract-Malt extract-Glucose (YMG) agar. *Clostridium tyrobutyricum* GIM1.262 (ATCC 25755) was obtained from Guangdong Microbiology Culture Center (GIMCC) and maintained at 4°C on Reinforced *Clostridium* Medium (RCM) agar.

### Media and seeds cultures

*C*. *acetobutylicum* seed culture method and medium preparation procedures for ABE fermentation were the same as those described in our previous study [23]. Seed culture of *S*. *cerevisiae* was conducted in 500 mL Erlenmeyer flasks containing 100 mL medium. *S*. *cerevisiae* seed was incubated in a rotating shaker at 200 r/min and 30°C for 24 h. The medium for yeast seed culture and cultivation in fermentor consisted of (in g/L): glucose 20, yeast extract 8.5, (NH_4_)_2_SO_4_ 1.3, MgSO_4_·7H_2_O 0.1, and CaCl_2_·2H_2_O 0.06. Seed culture of *C*. *tyrobutyricum* ATCC 25755 was conducted in 100 mL anaerobic bottles containing 50 mL medium. *C*. *tyrobutyricum* seed was incubated in a water bath and 37°C for 24 h. The medium for *C*. *tyrobutyricum* seed culture consisted of (in g/L): glucose 30, yeast extract 5.0, peptone 5.0, (NH_4_)_2_SO_4_ 3.0, K_2_HPO_4_ 1.5, NaCl 6.0, MgSO_4_·7H_2_O 0.6, FeSO_4_·7H_2_O 0.03, and pH 6.0.

### *S*. *cerevisiae* cultivation

*S*. *cerevisiae* was cultivated in a 5 L fermentor (Baoxing Co., Ltd., China) under the conditions of pH 6.0, air-aeration and 30°C, by transferring 10% (v/v) seed culture broth into the medium. The standard DO-Stat method [24] was adopted for feeding concentrated glucose solution (500 g/L) to ensure both glucose and ethanol concentrations in the broth at near zero level during feeding stage. The cultivation was carried out in parallel with ABE fermentations. When the biomass of *S*. *cerevisiae* reached about 20 g-DCW/L (DCW = 0.22×OD_600_), a small portion of broth was harvested, and then added into the ABE fermentation broth according to requirements.

### Butyrate fermentation

Butyrate fermentation by *C*. *tyrobutyricum* was conducted in the 7 L anaerobic fermentor. The fermentation medium was the same as the seed medium except the initial glucose concentration (60 g/L). The loading volume of fermentation medium was 4.0 L. The temperature and pH during fermentation were maintained at 37°C and 6.0, respectively. 10% (v/v) of *C*. *tyrobutyricum* inoculum was transferred into the fermentor and the broth was aerated with N_2_ for 10 min to remove the dissolved oxygen. When the residual glucose concentration in broth dropped below 10 g/L, about 80 mL of glucose solution (600 g/L) was fed into the broth to bring glucose concentration up to 25 g/L. The fermentation was ended at 60 h when butyrate concentration reached about 28.5 g/L. The butyrate fermentative supernatant was then collected and concentrated in a rotary vacuum evaporator at 80°C and 0.03 MPa, to a concentrated level of 50.0 g/L. The supernatant was filtrated (0.22 μm) for sterilization and then kept in a refrigerator at 4°C. The supernatant was used in ABE fermentation to replace the chemically synthesized butyrate according to the requirement.

### ABE fermentation

The corn flour (starch content about 70%, w/w) was obtained at local market. The medium was pretreated by adding a tiny amount of α-amylase (8 U/g-corn flour, heated in boiling water bath for 45 min) and then glucoamylase (120 U/g-corn flour, heated at 62°C for 60 min). Subsequently, the viscosity reduced medium was autoclaved at 121°C for 15 min. ABE fermentations were carried out at 37°C in 100 mL anaerobic bottles containing 50 mL fermentation medium (corn flour contents 15%, w/v). 10% (v/v) of *C*. *acetobutylicum* seed broth was transferred into the bottles. When *C*. *acetobutylicum* had been cultured for 24 h, *S*. *cerevisiae* broth harvested in the 5 L fermentor was added into the anaerobic bottles with the adding amount of 0.04–0.40 g-DCW/L-broth. According to the requirements, concentrated butyrate solution (pH adjusted to 6.0) amounted 4.0 g/L-broth was also added into the bottles at the same time. ABE fermentations were conducted in a 7 L anaerobic fermentor (Baoxing Co., Ltd., China) as well. A temperature controllable water bath was used to circulate warm water into the coil pipes settled inside the fermentor to maintain broth temperature at 37°C. The loading volume of fermentation medium was 3.5 L with the initial corn flour contents of 15% (w/v). 10% (v/v) of *C*. *acetobutylicum* inoculum was transferred into the fermentor and then the broth was sparged with N_2_ for 10 min. The pressure inside the fermentor was controlled in a range of 0.030–0.055 MPa by a manually adjustable pressure valve after self-generated gas began to evolve. According to the requirements, specified amounts of *S*. *cerevisiae* broth and concentrated butyrate solution (4.0 g/L-broth) were supplemented at 24 h when ABE fermentation entered solventogenic phase. Here, pH was regulated to 5.0 by adding 3 mol/L NaOH solution.

### Determination of the cell viability of *S*. *cerevisiae*

Viable *S*. *cerevisiae* cells were inoculated into the ABE fermentation broth at about 24 h. Then, the ABE broths were diluted in triplicate in a range of 10^7^∼10^8^ fold at each sampling time, plated on Yeast extract-Peptone-Dextrose (YPD) agar plates and incubated for 24 h at 30°C. *C*. *acetobutylicum* could not grow on YPD plate, the viable cell amounts of *S*. *cerevisiae* were measured by counting the colony forming units (CFU) on each plate.

### Analytical methods

The concentrations of solvents (butanol, acetone and ethanol) and organic acids (butyrate and acetate) were measured by a gas chromatograph (GC112A, Shanghai Precision Science Instrument Co., China) with iso-butanol as the internal standard [23]. The starch content of the broth was determined by the acid (HCl) hydrolysis method and the glucose concentration was calculated as 1.1-fold of starch concentration (g/L) [23]. The amino acids concentrations in the supernatant were determined by an HPLC (Agilent 1100, USA) [25]. The cellular morphological shapes of *C*. *acetobutylicum* and *S*. *cerevisiae* in pure cultivations and co-culturing system with/without exogenous butyrate addition were pictured by an Olympus BX53 camera (Olympus Corporation, Japan). In fermentor based fermentations, the evolved gas amount was measured by collecting the gas in a graduated tube filled with water every hour. The gas was firstly directed into two absorption bottles connected in-series filled with 6 mol/L NaOH solution, to determine H_2_ release amount within a period of 15 min. Then total evolved gas amount was measured in the remaining 45 min without passing through the absorption bottles. H_2_ and CO_2_ evolution rates (*r*_H2_, *r*_CO2_) were calculated by Eq 1.

$$r_{H2}=(A_{H2}\times 4)/L\;r_{CO2}=(A_{GAS}\times 1.33-A_{H2}\times 4)/L$$ (1)

where *A*_H2_ and *A*_GAS_ were H_2_ gas formation volume measured in the first 15 min and total gas evolved volume in the rest 45 min, respectively. *L* was the volume of ABE fermentation broth. *A*_H2_ and *A*_GAS_ were then converted into the unit of mol/L-broth/h (*r*_H2_ and *r*_CO2_) using the ideal gas equation (25°C, 1 atm). Concentrations of residual glucose, ethanol and acetone were smoothed by polynomial fitting with fermentation time as the independent variable, and their consumption/synthesis rates (*r*_GLC_, *r*_EtOH_ and *r*_ACE_, mol/L/h) at instant *T* were then determined by differentiating the fitted concentrations curves with regards to *T*. Those rate parameters (together with *r*_H2_ and *r*_CO2_) were then used for determining the theoretical NADH regeneration rate for butanol synthesis in *C*. *acetobutylicum* that will be described in the following section.

### Estimation of formation/consumption of NADH, glucose and ethanol in *C*. *acetobutylicum* in the co-culturing system

In ABE fermentation by solventogenic *Clostridia*, butanol synthesis and ratio are controlled by NADH (reductive power) availability or its regeneration rate [26, 27]. Butanol synthesis in ABE fermentation could be enhanced if the substrates (glycerol and mannitol) with more reductive power were used [28]. In our case, experimental analysis of intracellular NADH in *C*. *acetobutylicum* is very difficult because the cells concentration was very low and the cells mixed tightly with the solid starch particles or/and *S*. *cerevisiae* cells. As a result, we had to estimate glucose consumption rate (*r*_GLC_^CA^) and NADH production rate (*r*_NADH_^BtOH^) required for butanol synthesis in *C*. *acetobutylicum* for the co-culturing system, based on the measurable fermentation parameters and the reasonable assumptions. As both *C*. *acetobutylicum* and *S*. *cerevisiae* produce ethanol, a parameter *γ* was defined as the contribution ratio of ethanol synthesis by *S*. *cerevisiae* over the total ethanol produced.

Assumptions: 1) In standard ABE fermentation by *C*. *acetobutylicum*, the well-recognized stoichiometric mass balance equation of Eq 2 is generally applied [29], while ethanol synthesis by *S*. *cerevisiae* yields mass balance equation of Eq 3 (both in mole base).

$$GLC\to 0.63BtOH+0.315ACE+0.11EtOH+2.32CO_{2}+1.26H_{2}+0.32H_{2}O$$ (2)

$$GLC\to 2EtOH+2CO_{2}$$ (3)

2) The stoichiometric ratio of *r*_EtOH_/*r*_CO2_ (mole base) in *C*. *acetobutylicum* and *S*. *cerevisiae* metabolisms does not vary under the co-culturing operation mode, that is,

$$r_{EtOH}=r_{EtOH}^{CA}+r_{EtOH}^{SC}\;r_{CO2}=r_{CO2}^{CA}+r_{CO2}^{SC}$$ (4)

$$\frac{r_{EtOH}^{SC}}{r_{CO2}^{SC}}=\frac{\gamma \times r_{EtOH}}{r_{CO2}^{SC}}=1$$ (5a)

$$\frac{r_{EtOH}^{CA}}{r_{CO2}^{CA}}=\frac{(1-\gamma )r_{EtOH}}{r_{CO2}-r_{CO2}^{SC}}=\frac{(1-\gamma )r_{EtOH}}{r_{CO2}-\gamma \times r_{EtOH}}=\frac{0.11}{2.32}=0.047$$ (5b)

Here, *r*_EtOH_, *r*_EtOH_^CA^, *r*_EtOH_^SC^, *r*_CO2_, *r*_CO2_^CA^ and *r*_CO2_^SC^ represented total (apparent) ethanol production rate, ethanol synthesis rates by *C*. *acetobutylicum* and *S*. *cerevisiae*, total CO_2_ production rate, CO_2_ evolution rates by *C*. *acetobutylicum* and *S*. *cerevisiae*, respectively. The reasonability of the two assumptions will be discussed in the section of “Results and Discussions”. Combining Eq 4 and Eq 5, *γ* could be then calculated by Eq 6.

$$\gamma (T)=\frac{r_{EtOH}^{SC}}{r_{EtOH}}=\frac{r_{EtOH}(T)-0.047r_{CO2}(T)}{\left(1-0.047)r_{EtOH}(T\right)}=1.05\left(1-0.047\frac{r_{CO2}(T)}{r_{EtOH}(T)}\right)\;0\leq \gamma (T)\leq 1$$ (6)

where *r*_CO2_ and *r*_EtOH_ could be experimentally measured. *T* referred to the sampling time. Concentration of ethanol synthesized by *C*. *acetobutylicum* (EtOH^CA^) could be formulated by Eq 7, with *γ* = 0 representing pure ABE fermentation by *C*. *acetobutylicum*. Δ*T*, *N* and *MW*_EtOH_ were sampling interval, sample number and ethanol molecular weight, respectively.

$$\begin{array}{l} EtOH^{CA}(T)=MW_{EtOH}\sum_{T=1}^{N}r_{EtOH}^{CA}(T)\Delta T=MW_{EtOH}\sum_{T=1}^{N}\left((1-\gamma (T))r_{EtOH}(T)\Delta T\right)\;T=1,2,\ldots N\\ EtOH^{CA}(T)=EtOH(T)\;if\;\gamma =0 \end{array}$$ (7)

Based on the mathematical formula reported by our previous study [15], the glucose utilization rate (*r*_GLC_^CA^) and butanol synthesis oriented NADH utilization rate (*r*_NADH_^BtOH^) in *C*. *acetobutylicum* could be estimated by Eq 8 and Eq 9, using the calculated *γ*(*T*), as well as the measured parameters of *r*_GLC_, *r*_EtOH_, *r*_CO2_, *r*_H2_ and *r*_ACE_. In addition, the total glucose amount consumed by *S*. *cerevisiae* (GLC^SC^) could also be calculated by Eq 10, where *MW*_GLC_ represented the molecular weight of glucose.

$$r_{GLC}^{CA}(T)=r_{GLC}-r_{GLC}^{SC}=r_{GLC}-\frac{1}{2}r_{EtOH}^{SC}=r_{GLC}(T)-\frac{\gamma (T)}{2}r_{EtOH}(T)$$ (8)

$$\begin{array}{l} r_{NADH}^{BtOH}(T)=2r_{CO2}^{CA}\left(1-\frac{r_{ACE}}{r_{CO2}^{CA}}-\frac{1}{2}\frac{r_{H2}}{r_{CO2}^{CA}}\right)-2r_{EtOH}^{CA}\\ =2(r_{CO2}(T)-\gamma (T)\times r_{EtOH}(T))\times \\ \left(1-\frac{r_{ACE}(T)}{r_{CO2}(T)-\gamma (T)\times r_{EtOH}(T)}-\frac{1}{2}\frac{r_{H2}(T)}{\left(r_{CO2}(T)-\gamma (T)\times r_{EtOH}(T)\right)}\right)-2(1-\gamma (T))r_{EtOH}(T) \end{array}$$ (9)

$$GLC^{SC}=MW_{GLC}\sum_{T=1}^{N}\frac{\gamma (T)}{2}r_{EtOH}(T)\;T=1,2,\ldots N$$ (10)

## Results and Discussion

### Exogenous butyrate addition improving performance of pure ABE fermentation by promoting amino acids secretion and enhancing NADH regeneration rate

**Fig 1:**
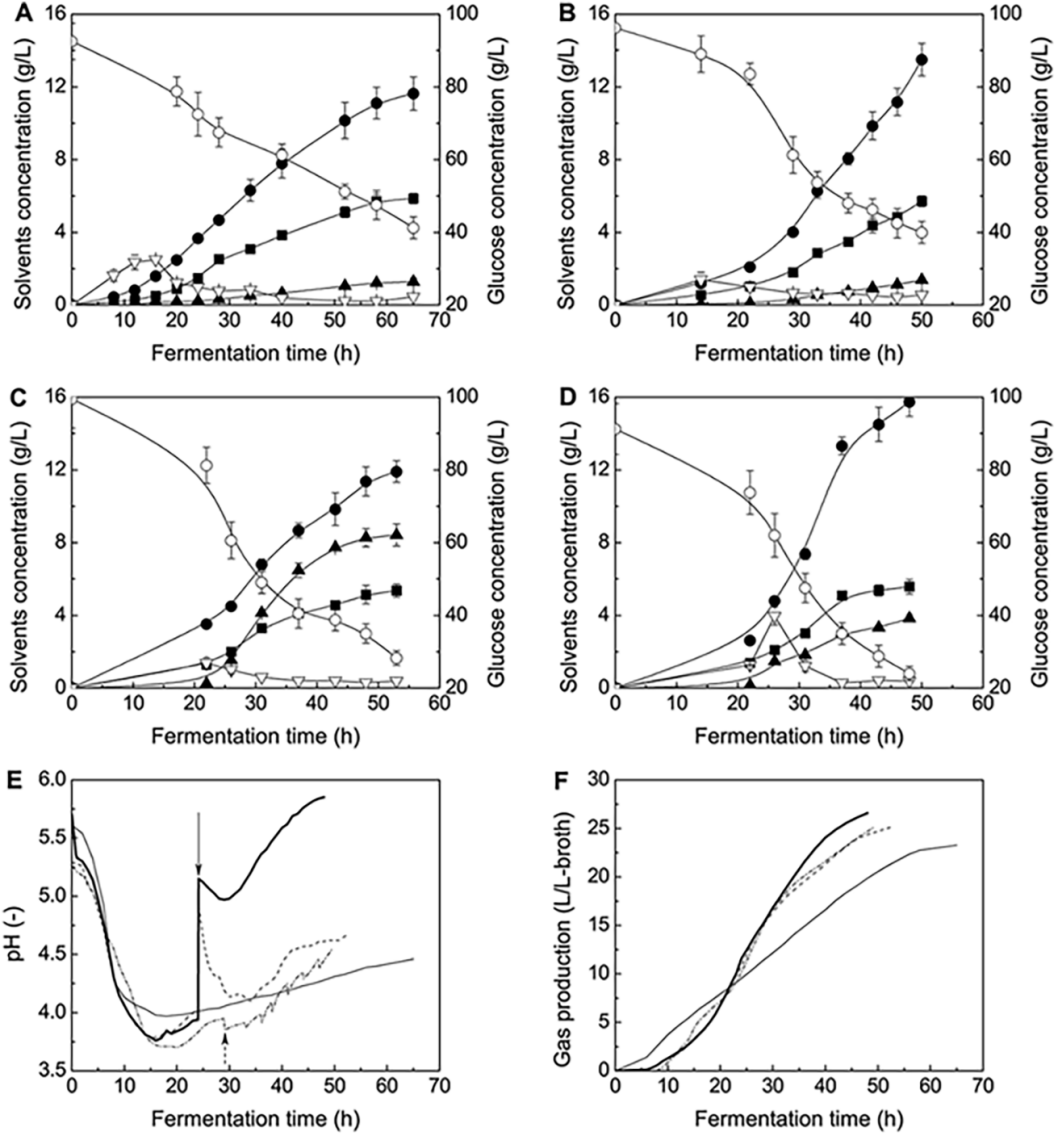
ABE fermentation performance with different operation strategies. **(A)** ABE fermentation by *C*. *acetobutylicum* (control, case #a). **(B)** ABE fermentation by *C*. *acetobutylicum* with exogenous butyrate addition (case #b). **(C)** ABE fermentation by co-culturing *C*. *acetobutylicum*/*S*. *cerevisiae* (case #c). **(D)** ABE fermentation by co-culturing *C*. *acetobutylicum*/*S*. *cerevisiae* in coupling with exogenous butyrate addition (case #d). ●: butanol; ■: acetone; ▲: ethanol; ▽: butyrate; ○: glucose. **(E)**-**(F)** Change patterns of pH and gas production with different operation strategies. Thin line: case #a (control); dash dot line: case #b; broken line: case #c; bold line: case #d. Dashed arrow: the instant of initiating the consecutive butyrate additions (case #b); solid arrow: the instant of adding *S*. *cerevisiae* culture broth (case #c) and *S*. *cerevisiae* culture broth/concentrated butyrate solution (case #d).

Butyrate is an important intermediate metabolite in ABE fermentation. In acidogenic phase, butyrate could accumulate to a level of up to 1.0∼2.0 g/L; and then it is gradually re-assimilated by *C*. *acetobutylicum* cells as a co-substrate during the solventogenic phase, the butyrate formation/re-assimilation closed-loop is thus formed. Butyrate is considered as an alternative substrate for ABE fermentation, which could regulate carbon fluxes in *C*. *acetobutylicum* metabolism and effectively increase butanol/carbon-sources conversion ratio [13, 30]. Our previous report also indicated that exogenous addition of a small amount butyrate resulted a higher butanol/acetone ratio [16]. Furthermore, exogenous butyrate addition could be considered as a kind of environmental stress to *C*. *acetobutylicum* survival, so that it may affect ABE fermentation by *C*. *acetobutylicum* in other aspects. Under the stress environment, some substances, such as amino acids, etc. which are beneficial for the cells survival, might be forced to be intracellular accumulated and extracellular secreted. We conducted ABE fermentation by *C*. *acetobutylicum* with exogenous butyrate addition in anaerobic bottles and fermentor, and compared the results with those of ABE fermentation without exogenous butyrate addition (control, case #a). The fermentation results were presented in Fig 1. In fermentor case, butyrate was consecutively fed in 7 times, with a total adding amount of 4.0 g/L-broth (case #b). Compared with control, butanol concentration increased from 11.63 g/L to 13.50 g/L, and butanol ratio rose from 0.62 to 0.66 (Fig 1A and 1B, Table 1). Actually, many literatures have reported the effectiveness of exogenously adding butyrate in improving ABE fermentation performance. The reports indicated that exogenous butyrate additions could alter metabolic flux distribution to direct more glucose into butanol synthesis route [13, 16] and the added butyrate could be used as a more efficient carbon source in presence of glucose because of no NADH consumption requirement in converting butyrate into butanol [14, 30, 31].

**Table 1:** Comprehensive performance comparison of ABE fermentation using different operation strategies.

Scale/ Run #	Operation strategies	Solvents concentration (g/L)				Ratios BtOH & B/A (g/g)	ABE prod. (g/L/h)	Glucose consumed (g/L)		Butyrate (g/L)	Yield (g/g-glucose)			
		BtOH	ACE	EtOH	ABE			Total	GLC^SC^		*Y*_BtOH_	*Y'*_BtOH_	*Y*_ABE_	*Y'*_ABE_
**0.1 L**	Control^1^	12.87	6.41	2.51	21.79	0.59/2.01	0.36	N/A	N/A	0.17	N/A	N/A	N/A	N/A
**0.1 L**	Butyrate addition^2^	15.29	5.67	2.24	23.20	0.66/2.70	0.39	N/A	N/A	0.43	N/A	N/A	N/A	N/A
**0.1 L**	Co-culture^3^	13.95	6.12	4.16	24.23	0.58/2.28	0.40	N/A	N/A	0.26	N/A	N/A	N/A	N/A
**0.1 L**	Co-culture+Butyrate^4^	16.34	5.50	3.50	25.34	0.64/2.97	0.42	N/A	N/A	0.17	N/A	N/A	N/A	N/A
**7 L/#a**	Control^1^	11.63	5.86	1.28	18.77	0.62/1.98	0.29	51.3	0.0	0.46	0.227	0.227	0.366	0.366
**7 L/#b**	Butyrate addition^2^	13.50	5.72	1.39	20.61	0.66/2.36	0.41	53.0	0.0	0.55	0.255	0.255	0.389	0.389
**7 L/#c**	Co-culture^3^	11.91	5.36	8.42	25.69	0.46/2.22	0.48	71.3	14.3	0.24	0.167	0.209	0.360	0.322
**7 L/#d**	Co-culture+Butyrate^4^	15.74	5.57	3.84	25.15	0.63/2.83	0.52	69.3	5.3	0.39	0.227	0.246	0.363	0.351
**7 L/#e**	Co-culture+Butyrate^5^	14.91	5.49	2.65	23.05	0.65/2.72	0.42	61.0	3.1	0.77	0.244	0.258	0.378	0.372

BtOH, ACE, EtOH and ABE refer to butanol, acetone, ethanol and total solvents; ratios of BtOH and B/A are butanol ratio over total ABE and butanol/acetone ratio; ABE prod. points to the total ABE productivity; GLC^SC^ refers to the glucose consumed amount by *S*. *cerevisiae*; Butyrate points to the final residual butyrate concentration. All yields (*Y*) are weight ratios (butanol-formed/glucose-consumed or ABE-formed/glucose-consumed, g/g); *Y'*_BtOH_ is the butanol yield on glucose consumed by *C*. *acetobutylicum*; *Y'*_ABE_ is the total ABE yield on glucose consumed by *C*. *acetobutylicum*. N/A: not available or measured.

^1^: Control refers to ABE fermentation by *C*. *acetobutylicum*.

^2^: Butyrate addition refers to ABE fermentation by *C*. *acetobutylicum* with exogenous butyrate addition.

^3^: Co-culture refers to ABE fermentation by co-culturing *C*. *acetobutylicum*/*S*. *cerevisiae*.

^4^: Co-culture+Butyrate refers toABE fermentation by co-culturing *C*. *acetobutylicum*/*S*. *cerevisiae* in couple with exogenous butyrate addition.

^5^: Co-culture+Butyrate refers to ABE fermentation by co-culturing *C*. *acetobutylicum*/*S*. *cerevisiae* integrated with exogenous butyrate fermentative supernatant addition (4.0 g-butyrate/L-broth).

On the other hand, many reports showed that, some amino acids, either self-generated or exogenously added, are favorable for butanol synthesis or cells survival in ABE fermentation by *C*. *acetobutylicum*, which would lead to the performance improvement. A study using a chemically defined medium, showed that exogenously adding aromatic family amino acids (phenylalanine and tyrosine) has positive effects on butanol synthesis (butanol concentration rose from 12.0 g/L to 14.2 g/L) and cells growth (maximum biomass increased from 3.44 g/L to 4.57 g/L) [32]. Another report indicated that aspartic family amino acids (lysine and methionine) could up-regulate butanol synthesis [33]. We also added aromatic and aspartic family amino acids, as well as their combinations, into the anaerobic bottles based ABE fermentation broth when it entered into solventogenic phase at 24 h, attempting to improve ABE fermentation performance. In our case, the largest improvement in ABE fermentation occurred when adding aspartic family amino acids (0.80 g/L-broth lysine and 0.20 g/L-broth methionine), butanol concentration and butanol/acetone ratio increased from 11.59 g/L to 12.98 g/L and 1.91 to 2.27, respectively. However, improvement in ABE fermentation performance by exogenously adding those amino acids was very limited.

**Fig 2:**
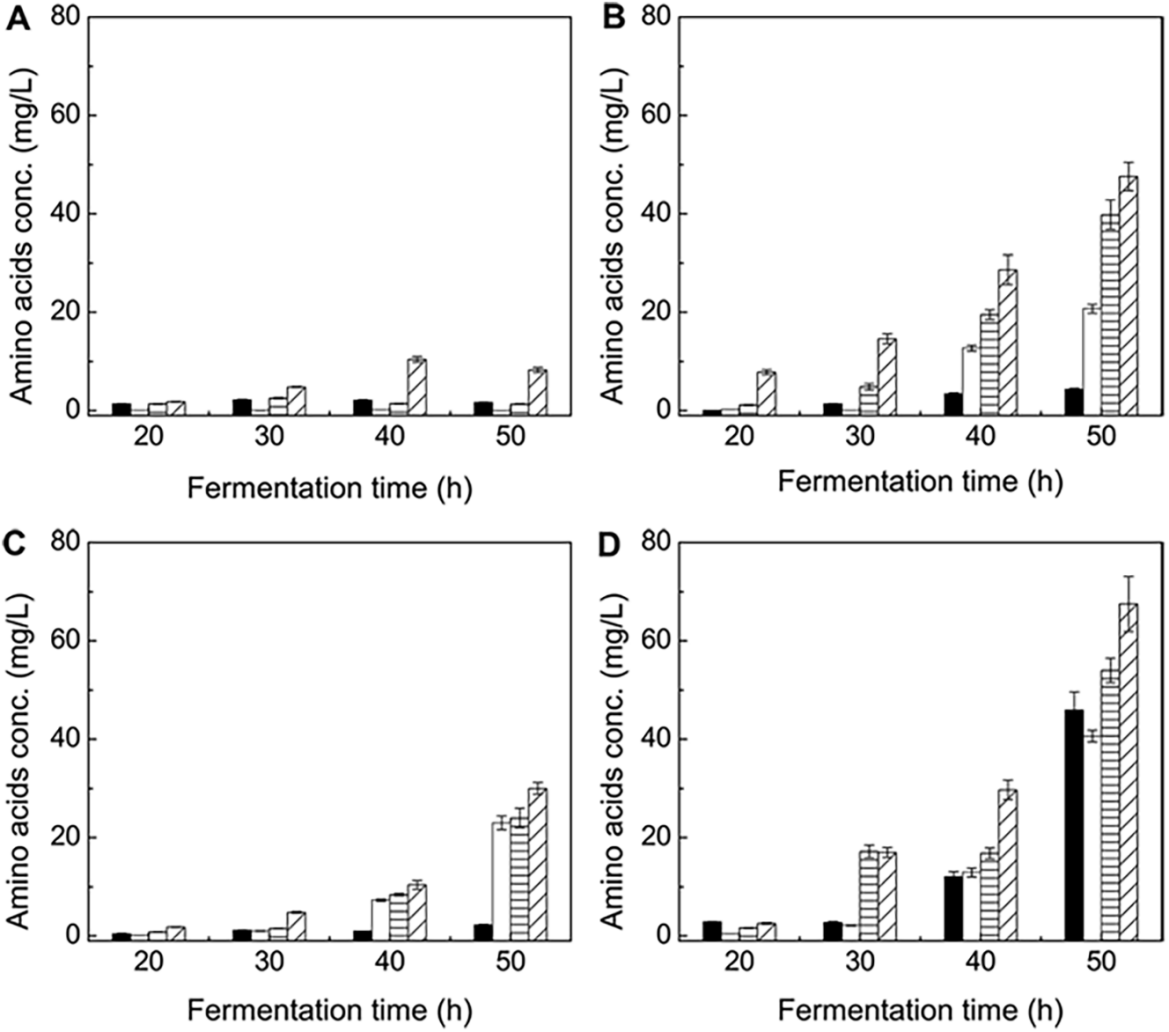
Amino acids secretion patterns with different operation strategies. **(A)** ABE fermentation by *C*. *acetobutylicum* (control, case #a). **(B)** ABE fermentation by *C*. *acetobutylicum* with exogenous butyrate addition (case #b). **(C)** ABE fermentation by co-culturing *C*. *acetobutylicum*/*S*. *cerevisiae* (case #c). **(D)** ABE fermentation by co-culturing *C*. *acetobutylicum*/*S*. *cerevisiae* in coupling with exogenous butyrate addition (case #d). Black: lysine; white: methionine; horizontal shadow: phenylalanine; slashed shadow: tyrosine.

By exogenously adding 4.0 g/L-broth butyrate into ABE fermentation broth in 7 L anaerobic fermentor, first of all, we found that the “favorable” amino acids of phenylalanine, tyrosine and methionine (expect lysine) could be secreted into the broth at certain amounts, while the secretions actually reflected their accumulations in *C*. *acetobutylicum*. Fig 2A and 2B depicted the secretion patterns of the four amino acids in the ABE fermentations with/without butyrate addition. With butyrate addition, those amino acids except lysine gradually secreted and their concentrations increased continuously, while the amino acids concentrations remained unchanged in control case. Based on the results, we speculated that, exogenous butyrate addition stimulated intracellular accumulation of the three amino acids favorable for butanol synthesis and cells survival. Alsaker et al. studied the metabolic mechanism of the anaerobe *C*. *acetobutylicum* based on gene-expression-based analysis, when implementing butanol and butyrate stress [34]. Their results revealed that some genes, which encoded phenylalanine and tyrosine biosynthesis, were differentially upregulated by butanol and butyrate stress. This conclusion was consistent with our findings of the amino acids secretion patterns in case of exogenously adding butyrate (case #b).

**Fig 3:**
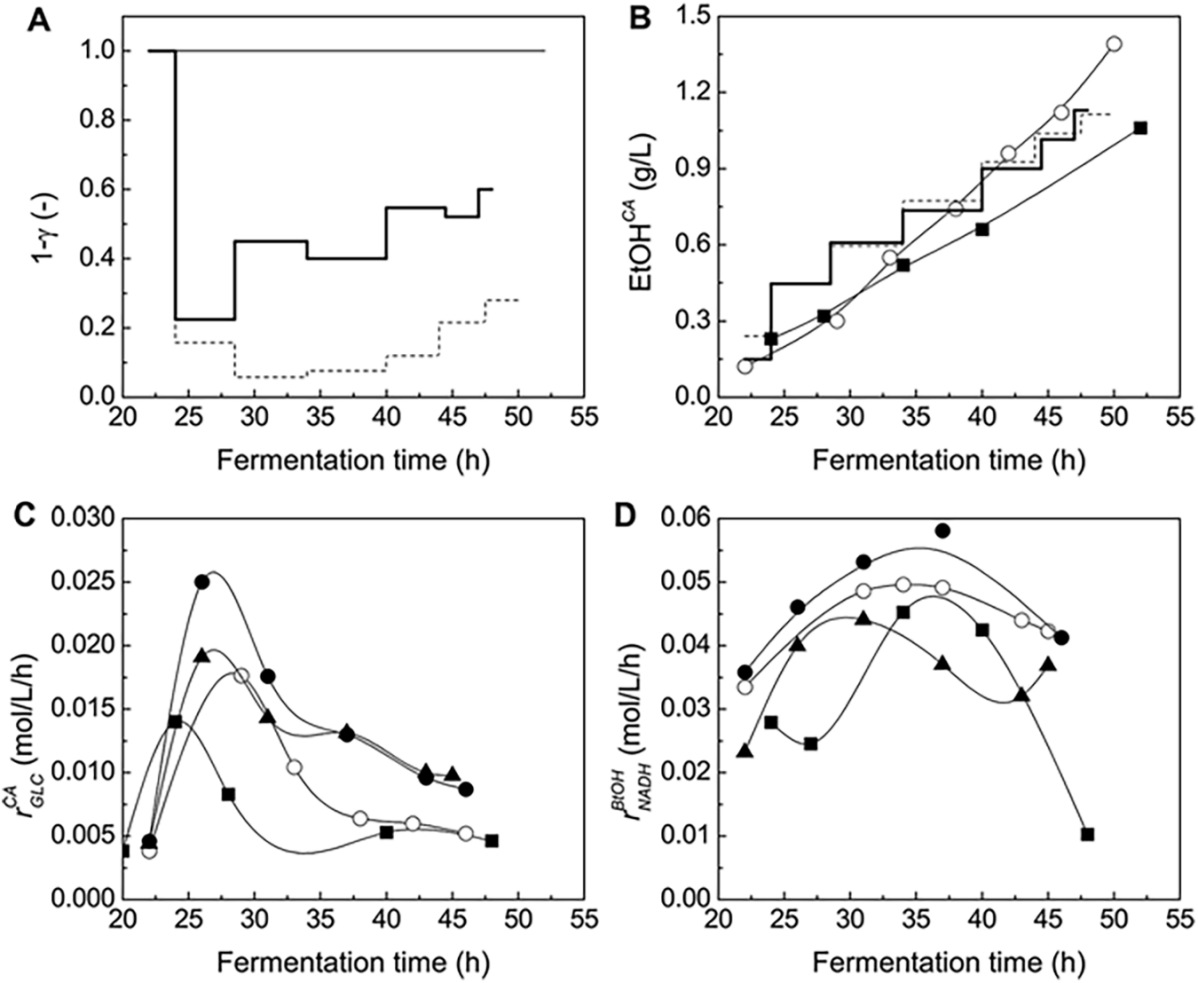
Theoretical analysis results of the major secondary parameters associated with ABE fermentation performance when using different operation strategies. **(A)** Contribution ratio of ethanol production by *C*. *acetobutylicum*. Thin line: ABE fermentation by *C*. *acetobutylicum* (control, case #a) and ABE fermentation by *C*. *acetobutylicum* with exogenous butyrate addition (case #b); broken line: ABE fermentation by co-culturing *C*. *acetobutylicum*/*S*. *cerevisiae* (case #c); bold solid line: ABE fermentation by co-culturing *C*. *acetobutylicum*/*S*. *cerevisiae* with butyrate addition (case #d). **(B)** Ethanol synthesis by *C*. *acetobutylicum* (EtOH^CA^). ■: case #a (control); ○: case #b; broken line: case #c; bold solid line: case #d. **(C)-(D)** Change patterns of *r*_GLC_^CA^ and *r*_NADH_^BtOH^. ■: case #a (control); ○: case #b; ▲: case #c; ●: case #d.

A report indicated that the exogenously added butyrate (molecule form) could penetrate into the *C*. *acetobutylicum* cells, and then the dissociation equilibrium of CH_3_CH_2_CH_2_COOH ↔ CH_3_CH_2_CH_2_COO^−^+ H^+^ was formed intracellular inducing the formation of excessive proton H^+^ [35]. As a result, exogenous butyrate addition might not only stimulate amino acids secretions but also enhance the regeneration rate of NADH (*r*_NADH_^BtOH^, calculated by the prediction model Eq 9, *γ* = 0) required for butanol synthesis in *C*. *acetobutylicum*. As shown in Fig 3D, *r*_NADH_^BtOH^ in the case of exogenous butyrate addition (case #b) was about 10–50% higher than that of control (case #a) during the solventogenesis phase, the enhanced *r*_NADH_^BtOH^ was also responsible for the enhanced butanol concentration and ratio.

Therefore, the synergetic effect of amino acids intracellular accumulation, enhanced intracellular *r*_NADH_^BtOH^, as well as metabolic regulation by exogenous butyrate addition, contributed to ABE fermentation performance improvement co-operatively. However, the increments in butanol concentration and ratio were limited with this butyrate addition strategy. Therefore, further advanced fermentation strategies were explored.

### The possibility of improving ABE fermentation by co-culturing *C*. *acetobutylicum*/*S*. *cerevisiae* by investigating interactions and dynamics of *S*. *cerevisiae* and *C*. *acetobutylicum* under each stress environments

First of all, we speculated that *S*. *cerevisiae* has the ability to survive under its in-favorable conditions (high temperature, high butanol/butyrate concentrations, strict anaerobic, etc.) by secreting some substances, such as amino acids, etc. to resist the stress environment. If the amino acids secreted by *S*. *cerevisiae* could be assimilated or utilized by *C*. *acetobutylicum* to protect these cells against the high butanol concentration in ABE fermentation, then ABE fermentation performance improvement could be expected.

**Fig 4:**
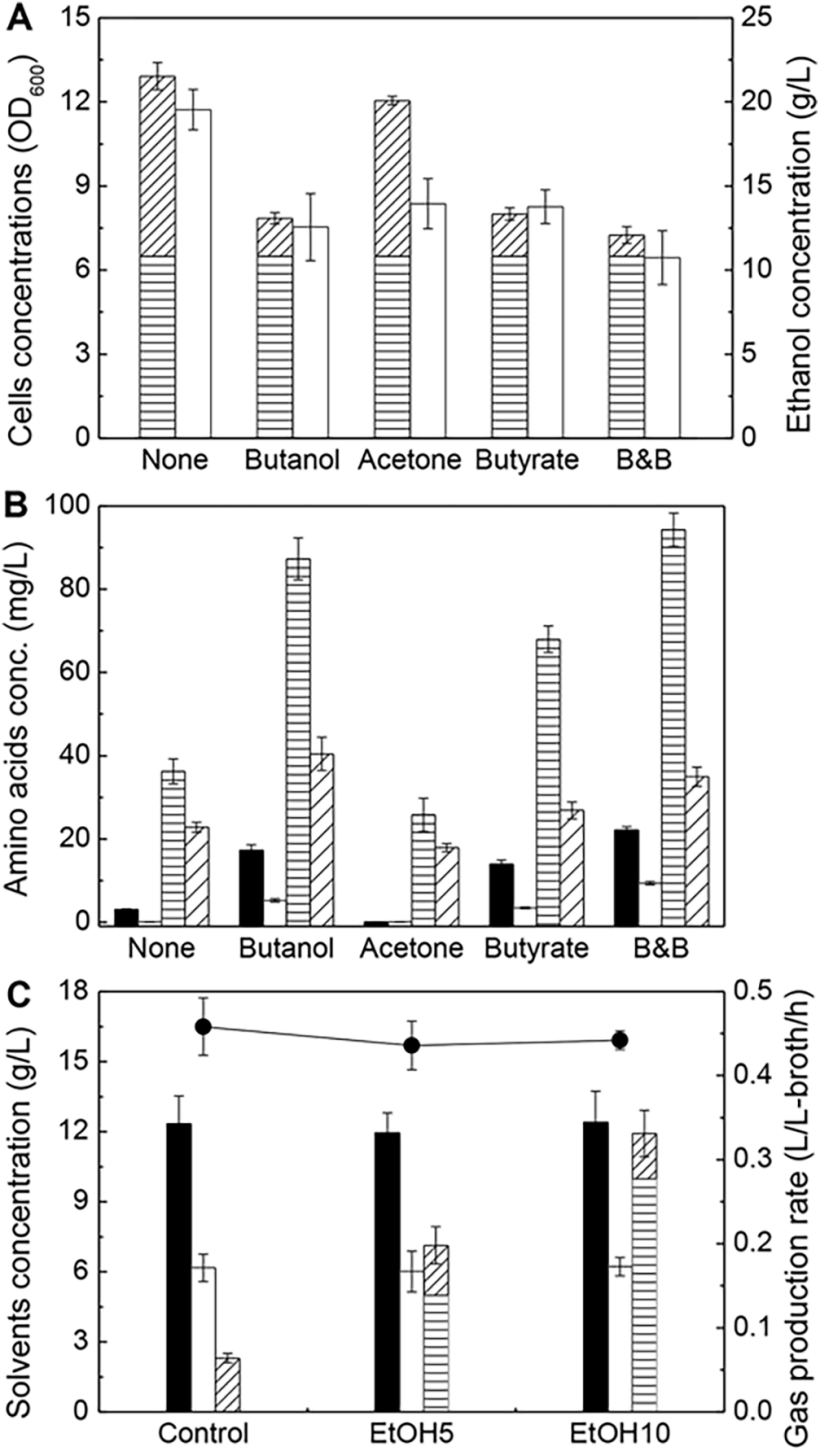
Investigation of interactions and dynamic behaviors of *S*. *cerevisiae* and *C*. *acetobutylicum* under each stress environments. **(A)** Cells growth and ethanol synthesis in *S*. *cerevisiae* cultivation. Horizontal shadow: Initial cells concentration (OD_600_); slashed shadow: increased cells concentrations (OD_600_); white: ethanol concentration. **(B)** The concentrations of the four amino acids favorable for *C*. *acetobutylicum* survival and butanol synthesis in *S*. *cerevisiae* cultivation. Black: lysine; white: methionine; horizontal shadow: phenylalanine; slashed shadow: tyrosine. None and B&B referred to the cases of no any ingredients additions and simultaneously adding butanol and butyrate, respectively. **(C)** Impacts of exogenous ethanol addition on ABE fermentation by *C*. *acetobutylicum*. Black: butanol; white: acetone; slashed shadow: ethanol produced by *C*. *acetobutylicum*; horizontal shadow: added ethanol; ●: gas production amount. EtOH5 and EtOH10: 5 g-ethanol/L and 10 g-ethanol/L were added. **(A)**-**(B)** *S*. *cerevisiae* seeds were incubated in a rotating shaker at 200 r/min and 30°C for 24 h and then transferred into the anaerobic bottles. The cultivation continued for 36 h. **(C)** The fermentations ended at 60 h, ethanol was added at 24 h.

The environments of ABE fermentation by *C*. *acetobutylicum* is considered to be a stress to *S*. *cerevisiae*. The doubts may be raised with regards to the yeast survival ability and the lose of the expected functions under very high butanol concentration (>10 g/L). Knoshaug et al. investigated the butanol tolerance in a selection of microorganisms, which indicated that *S*. *cerevisiae* could tolerate 1–2% (w/v) butanol environment [36]. Another report also revealed that *S*. *cerevisiae* could still maintain about 10% relative growth under 2% butanol environment [37]. Furthermore, butyrate, either self-generated or exogenously added may further generate the stress environment to *S*. *cerevisiae*. Therefore, we conducted the following preliminary *S*. *cerevisiae* cultivation experiments in 100 mL anaerobic bottles, under the environments of strictly anaerobic, 37°C and glucose sufficiency, by intently adding butanol (B, 10 g/L), acetone (5 g/L), butyrate (4 g/L) and butanol/butyrate (B&B, 10 g/L butanol + 4 g/L butyrate) to simulate the interactions and dynamic behaviors of *S*. *cerevisiae* under ABE fermentation environments. Fig 4A and 4B showed the cultivation results, including *S*. *cerevisiae* growth, ethanol synthesis, and the secretions of the four amino acids favorable for *C*. *acetobutylicum* cells survival of and butanol synthesis in *C*. *acetobutylicum*. *S*. *cerevisiae* growth were severely inhibited but ethanol synthesis was not much repressed, under the conditions of exogenously adding butanol, butyrate and butanol/butyrate. In these cases, cells growth still continued and did not stop completely as shown in Fig 4A. The secretion amounts of the four “favorable” amino acids were effectively enhanced (Fig 4B). On the other hand, the environments of high ethanol concentration (> 10 g/L) created by *S*. *cerevisiae* cultivation could be considered to be a stress to ABE fermentation by *C*. *acetobutylicum* in turn. Fig 4C showed the impacts of exogenous ethanol addition on ABE fermentation performance including the synthesis amounts of butanol/acetone/ethanol and total gas production amount. The results revealed that ABE fermentation performance of EtOH5 (5 g/L ethanol addition) and EtOH10 (10 g/L ethanol addition) was comparable to that of control. Therefore, it could be concluded that ethanol accumulation up to 10 g/L could not deteriorate the metabolic viability of *C*. *acetobutylicum* and its abilities of synthesizing butanol, acetone and ethanol.

### Effect of *C*. *acetobutylicum*/*S*. *cerevisiae* co-culturing system on ABE fermentation

Based on the results of the above mentioned preliminary experiments, a *C*. *acetobutylicum*/*S*. *cerevisiae* co-culturing system was proposed, which might potentially enhance ABE fermentation performance in the following three aspects: 1) the amino acids secreted by *S*. *cerevisiae* might be assimilated by *C*. *acetobutylicum* for more efficient butanol synthesis; 2) when *C*. *acetobutylicum* co-exists with *S*. *cerevisiae*, *C*. *acetobutylicum* has to compete with the yeast on substrate utilization to increase its substrate consumption rate, leading to a higher production rate (*r*_NADH_^BtOH^) of NADH required for butanol synthesis in *C*. *acetobutylicum* metabolism; 3) Due to the facts that the yeast and *Clostridia* strains were competing on substrate utilizations and consuming substrate simultaneously, glucose consumed amounts might be largely increased. If some of the extra consumed glucose could be utilized by *C*. *acetobutylicum* and butanol conversion yield from glucose did not vary, then extra butanol production (concentration) would be expected.

Theoretically, *S*. *cerevisiae* growth and the associated ethanol synthesis could be suitably repressed by certain manner to ensure *C*. *acetobutylicum* metabolism to be dominated in the co-culturing system. The viable *S*. *cerevisiae* adding amount and time, as well as temperature of the co-culturing system were optimized in anaerobic bottles by orthogonal design experiments. The preliminarily optimal conditions of temperature, the yeast adding amount and instant were determined as 37°C, 0.2 g-DCW/L-broth and 24 h after initiating the ABE fermentations. The major results of fermentor based ABE fermentation by co-culturing *C*. *acetobutylicum*/*S*. *cerevisiae* (case #c) and the comparisons with control (case #a) were shown in Figs 1–3 and Table 1. In this case (case #c), butanol concentration only slightly increased from 11.63 g/L to 11.91 g/L and butanol ratio dropped from 0.62 to 0.46 (though butanol/acetone ratio rose from 1.98 to 2.22), this was because final ethanol concentration accumulated to a very high level of 8.42 g/L and ethanol even became the secondly ranked end-product in ABE fermentation. In this case, the metabolic activity of *S*. *cerevisiae* was too high, and the metabolic competition between *C*. *acetobutylicum* and *S*. *cerevisiae* was not properly controlled. This actually deteriorated the ABE fermentation performance.

Similar as the case of pure ABE fermentation by *C*. *acetobutylicum* with exogenous butyrate addition (case #b), the three “desirable” amino acids (methionine, phenylalanine and tyrosine) also gradually secreted and their concentrations increased continuously, but almost no lysine secretion was observed as well (Fig 2C). In case #c, concentrations of the four amino acids were lower than those of case #b. We speculated that the secreted amino acids in case #c mainly originated from *S*. *cerevisiae*, as *C*. *acetobutylicum* did not suffer any other extra environmental stress except the higher ethanol accumulation. As shown in Fig 4C, *C*. *acetobutylicum* metabolic activities and ABE synthesis ability could not be deteriorated under ethanol stress environment if its concentration does not exceeded 10 g/L, thus 8.42 g/L ethanol accumulation in case #c would not stimulate the extracellular amino acids secretions. In case #b, the higher amino acids secretion concentrations (Fig 2B) implied a higher intracellular amino acids accumulation in *C*. *acetobutylicum*, which led the higher butanol concentration and ratio in turn. The secreted amino acids concentrations in case #c were higher than those of control (case #a) but lower than those of case #b. There was a possibility that, some of the secreted amino acids could enter into *C*. *acetobutylicum* cells due to the existence of extracellular-intracellular amino acids concentration gradient, but this was not enough to create equivalently enriched intracellular amino acids environment as that in case #b. As a result, we came to a conclusion that, the amino acids secreted by the yeast in the co-culturing system could not improve ABE fermentation performance.

**Fig 5:**
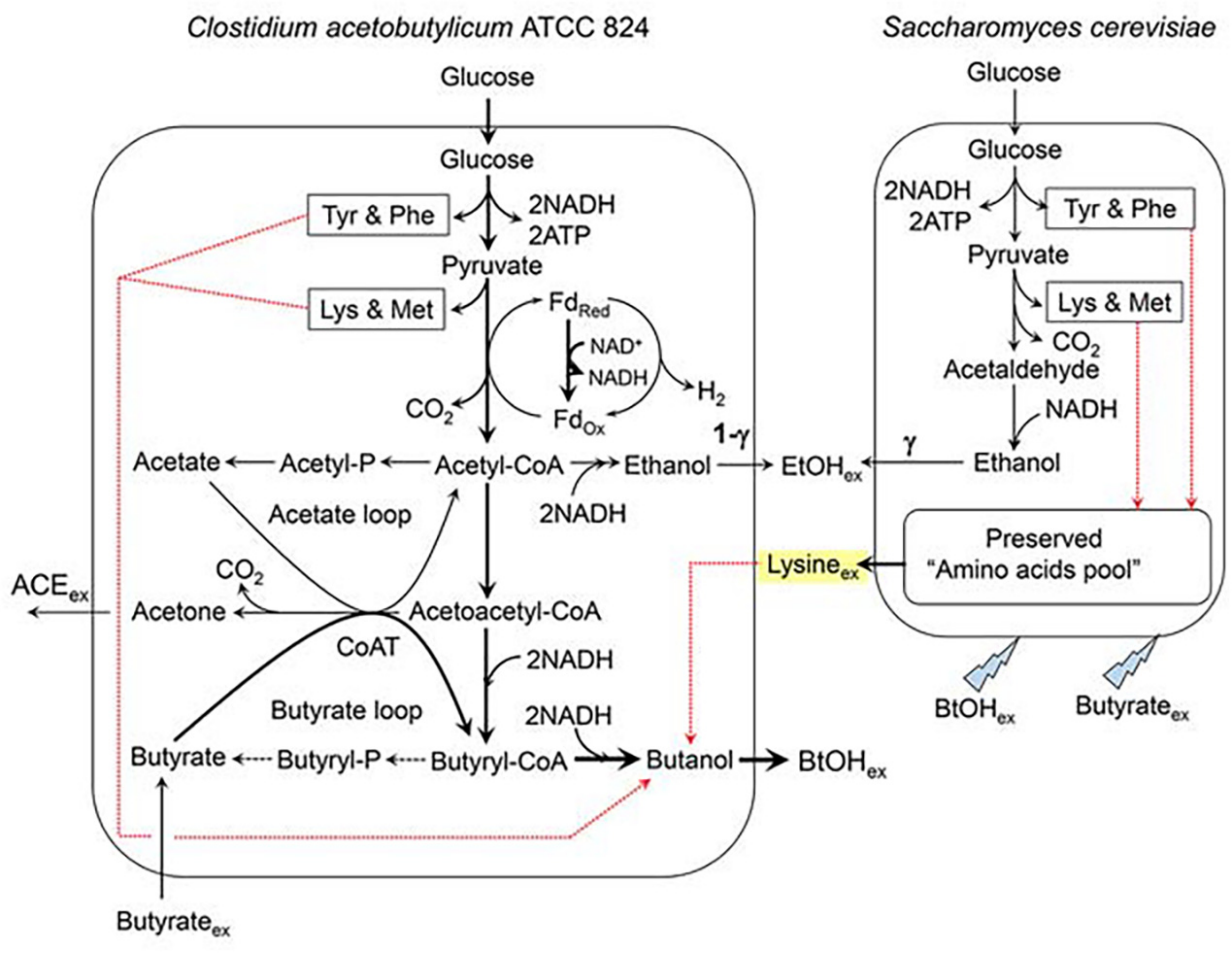
Simplified metabolic network map indicating *C*. *acetobutylicum* and *S*. *cerevisiae* metabolisms. Bold solid lines: enhanced metabolic fluxes; broken lines: weakened metabolic fluxes; dotted red lines: accumulation/secretion and assimilation/utilization directions of the amino acids favorable for butanol synthesis by *C*. *acetobutylicum* and *C*. *acetobutylicum* survival.

As mentioned above, in the co-culturing system, the *Clostridia* and yeast strains would compete on substrate utilizations and thus increase substrate consumption amounts. Certain part of the extra consumed substrate would be utilized by *C*. *acetobutylicum* for possible butanol and ABE productions enhancement. At the same time, intracellular NADH required for butanol synthesis could also be over-produced, as all of NADH is generated in the glycolysis route in *C*. *acetobutylicum* metabolism. Therefore, we attempted to use the previously described mathematical model (Eq 2–Eq 10) to estimate ratios of the extra substrate consumed by *C*. *acetobutylicum* and by *S*. *cerevisiae*, as well as the NADH regeneration rate *r*_NADH_^BtOH^ in *C*. *acetobutylicum*. This will definitely help us to find out the problems in the co-culturing system and to explore the ways to solve the problems. Here, a simplified metabolic network map covering both *C*. *acetobutylicum* and *S*. *cerevisiae* metabolisms was summarized in Fig 5.

As shown in Fig 1 and Table 1, total glucose consumption amount largely increased from 51.3 g/L to 71.3 g/L by co-culturing the two strains. Using the proposed mathematical model, the primary secondary fermentation parameters in the co-culturing system (case #c) could be calculated, and the parameters included glucose comsumption rate *r*_GLC_^CA^, NADH regeneration rate *r*_NADH_^BtOH^ and ethanol synthesis amount EtOH^CA^ in/by *C*. *acetobutylicum*, as well as the extra glucose amount consumed by *S*. *cerevisiae* GLC^SC^. As shown in Fig 3, *r*_GLC_^CA^ was effectively increased as compared with those of pure ABE fermentations by *C*. *acetobutylicum* with/without exogenous butyrate addition (case #a, control; case #b). However, the enhancement in *r*_NADH_^BtOH^ was limited and *r*_NADH_^BtOH^ was even lower than that of case #b. As for ethanol formation, *S*. *cerevisiae* contributed 80–90% (average *γ*≈0.8∼0.9) of the total ethanol synthesized amounts. Furthermore, the model estimated that, 72% of the extra consumed glucose was used by *S*. *cerevisiae* to over-produce ethanol while only 28% of the extra consumed glucose was utilized for ABE solvents production by *C*. *acetobutylicum*. Therefore, the ABE fermentation strategy of co-culturing *C*. *acetobutylicum*/*S*. *cerevisiae* could largely increase total glucose consumption amount and effectively increase *r*_GLC_^CA^, however, only 28% of the extra consumed glucose was used by *acetobutylicum* leading to a very limited *r*_NADH_^BtOH^ enhancement in turn. This strategy failed to improve the entire ABE fermentation performance.

**Fig 6:**
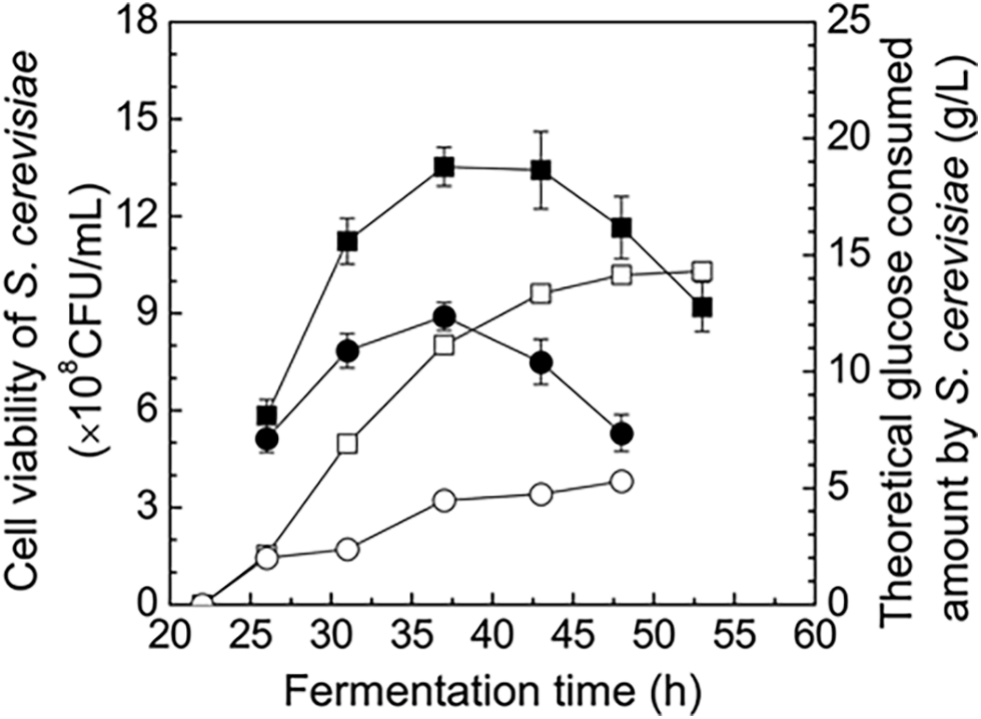
*S*. *cerevisiae* cells viability and theoretical glucose amount consumed by *S*. *cerevisiae* in the proposed co-culturing system. ○ & ●: cell viability of *S*. *cerevisiae* in the co-culturing system without/with butyrate addition; □ & ■: theoretical glucose amounts consumed by *S*. *cerevisiae* in the co-culturing system without/with butyrate addition.

It must be addressed that two assumptions were made when developing the prediction model (Eqs 2–10) to estimate the primary secondary parameters in evaluating the co-culturing based ABE fermentations. The two assumptions (Eq 5a and Eq 5b) were the stoichiometric coefficients of ethanol and CO_2_ formation rates in *S*. *cerevisiae* and *C*. *acetobutylicum*, and they should be reasonable under the following technical backgrounds or aspects. First of all, under the strict anaerobic ABE fermentation environment, almost 100% of glucose could only be consumed via the metabolic route of GLC → 2 EtOH + 2 CO_2_ (Eq 3) in *S*. *cerevisiae*, which was supported by the facts that the yeast growth ceased (Fig 4A and Fig 6) and only a tiny amount of glucose was used for cell maintenance. This tiny amount of glucose could be completely ignored in developing the prediction model. Secondly, in the standard ABE fermentation by *C*. *acetobutylicum*, the well-recognized stoichiometric mass balance equation (Eq 2) is generally applied, with the butanol/acetone/ethanol mole ratio of 6:3:1 [29]. There would be less doubt regarding the first assumption (Eq 5a), but argument might be raised on the second assumption (Eq 5b), which is if the *r*_EtOH_^CA^/*r*_CO2_^CA^ ratio of 0.0474 could still be held in the cases of co-culturing based ABE fermentations. We used ethanol and CO_2_ formation rates to evaluate the co-culturing system based on the following reasons: 1) ethanol and CO_2_ are the only two measurable metabolites produced by both *C*. *acetobutylicum* and *S*. *cerevisiae*, therefore it is reasonable to utilize the two parameters to model the co-culturing system; 2) glycolysis route (GLC → acetyl-CoA) in *C*. *acetobutylicum* metabolism is quite robust against environmental disturbances as no branched nodes exist in the route, and a couple of literatures [11, 29] reported that the ethanol mole ratio over total ABE solvents could be solidly maintained at 0.1 (0.11/(0.63+0.315+0.11)≈0.1, Eq 2) by different but traditional operation modes without gene manipulation involvements; 3) in pure and co-culturing based ABE fermentations, 100% H_2_ is from the electron transport shuttle system in *C*. *acetobutylicum* metabolism (Fig 5). Although the higher intracellular butyrate concentration would induce excessive production of proton H^+^, the excessive H^+^ could be consumed in the following two routes: NAD + H^+^ → NADH and 2H^+^ → H_2_ under the catalysis of hydrogenase [35]. If hydrogenase activity is not affected by the co-culturing system or/and butyrate addition (no reports were found yet regarding this), in both cases H_2_ would still be produced with the fixed stoichiometric coefficient of 1.26 (Eq 2), and the excessive H^+^ could be consumed in the route of NAD + H^+^ → NADH to over-produce NADH which is actually beneficial for butanol synthesis. In the case of existing large variations in butanol/acetone ratio (mole B/A, for example B/A largely varied from 2.0 to 3.0) which may associate with using the co-culturing system or/and adding butyrate, the stoichiometric coefficients variations in Eq 2 could be re-calculated by re-balancing the elements of C, H and O. Under this condition, *r*_EtOH_^CA^/*r*_CO2_^CA^ ratio and ethanol ratio over total ABE in Eq 2 only vary in the very narrow ranges (*r*_EtOH_^CA^/*r*_CO2_^CA^: 0.0469∼0.0474; ethanol ratio: 0.104∼0.107). In addition, similar as the yeast metabolism, when ABE fermentation entered the solventogenesis phase, the cell growth also stopped and only very little amount of glucose is consumed on cells maintenance as well. As a result, we believed that the two assumptions (Eq 5a & Eq 5b) are reasonable and robust enough in handling and predicting the behaviors of ABE fermentations with possible/large variations in butanol/acetone ratio.

### Novel fermentation strategy of co-culturing *C*. *acetobutylicum*/*S*. *cerevisiae* in coupling with exogenous butyrate addition significantly improving ABE fermentation performance

In *C*. *acetobutylicum* metabolism, a portion of the exogenously added butyrate could be directly converted into butanol via the metabolic route of butyrate → butyryl-CoA → butanol (intracellular) → BtOH_ex_ (butanol in supernatant) as shown in Fig 5. However, this metabolic route would not work without the existence of glucose, because 1 mol butyrate would consume 2 mol NADH to produce 1 mol butanol [13]. The required NADH has to be supplied by the glycolysis route in *C*. *acetobutylicum* metabolism. *C*. *acetobutylicum* has a complicated α-amylase and glucoamylase secretion structure to gradually hydrolyze the corn starch into glucose first, and then ABE synthesis is carried out by the hydrolyzed glucose. On the other hand, the pure co-culturing system could significantly increase total glucose consumption amount. *S*. *cerevisiae* has stronger glucose utilization ability than that of *C*. *acetobutylicum*, the hydrolyzed glucose was utilized by *S*. *cerevisiae* in priority leading to a very low ratio of extra substrate consumed by *C*. *acetobutylicum* (28%). In principle, the exogenously added butyrate has to be quickly used out to relieve the stress pressure of *C*. *acetobutylicum* cells against the high butyrate concentration environment, thus the added butyrate could be considered as an “inducer” for accelerating glucose consumption rate in *C*. *acetobutylicum* to satisfy the requirements of the stress relieve and butyrate bio-conversion. In this case, most of the extra consumed glucose might be directed into ABE production by *C*. *acetobutylicum*, significant enhancement in ratio of extra substrate consumed by *C*. *acetobutylicum* and intracellular NADH regeneration rate *r*_NADH_^BtOH^ could be expected. Based on the above speculation, a novel ABE fermentation strategy integrating *C*. *acetobutylicum*/*S*. *cerevisiae* co-culturing system with exogenous butyrate stress was thus proposed.

Table 1 and Fig 1D showed the ABE fermentation performance when implementing the newly proposed strategy. In anaerobic bottles case, both butanol concentration and butanol/acetone ratio reached the highest levels of 16.34 g/L and 2.97, while the total solvents concentration reached the highest level of 25.34 g/L as well. In 7 L fermentor, final butanol concentration and butanol/acetone ratio also reached the highest levels of 15.74 g/L and 2.83, which were 35% and 43% higher than those of control respectively. Furthermore, the total solvents (ABE) concentration and productivity also increased from 18.77 g/L to 25.15 g/L and 0.29 g/L/h to 0.52 g/L/h, respectively (Table 1). Final butyrate concentration declined to a low level of 0.39 g/L, indicating the added butyrate was mostly assimilated and utilized by *C*. *acetobutylicum* cells as a supplementary carbon source. With the novel fermentation strategy, ABE fermentation performance was significantly improved.

This novel ABE fermentation strategy (case #d) incorporated the advantages of *C*. *acetobutylicum*/*S*. *cerevisiae* co-culturing and exogenous butyrate addition simultaneously. With this strategy, intracellular accumulations of the amino acids favorable for *C*. *acetobutylicum* cells survival and butanol synthesis, as well as NADH required for butanol synthesis in *C*. *acetobutylicum* were largely enhanced. As shown in Figs 2D and 3D, secretion amounts of the four “favorable” amino acids and *r*_NADH_^BtOH^ were at the highest levels simultaneously among the four fermentations. For the amino acids, methionine, phenylalanine and tyrosine could be secreted from both *C*. *acetobutylicum* and *S*. *cerevisiae* (Fig 2B–2D), the total secretions were roughly the sum of those obtained in cases #b and #c. Interestingly, by adopting the novel fermentation strategy, lysine secretion was extremely enhanced for 27.5-fold (from 1.67 mg/L to 45.9 mg/L, Fig 2D). A report indicated that up-regulating the synthesis of lysine-specific permease was extremely important in protecting *C*. *acetobutylicum* cells under high butanol concentration environment [33]. We could not identify lysine secretion was originated from *C*. *acetobutylicum*, or from *S*. *cerevisiae*, or from both of them. Even for the most indirect case, that is, lysine was secreted from *S*. *cerevisiae*, such a huge amount of secreted lysine is quite possible to be assimilated into and utilized by *C*. *acetobutylicum*. The similar phenomenon was also observed in ABE fermentation by co-culturing *C*. *acetobutylicum*/*S*. *cerevisiae* integrated with exogenous acetate addition (4.0 g/L-broth). In this case, lysine was extensively secreted as well, and up to 25.3 mg/L lysine was released which was about 15.0-fold higher than those of cases #a, #b and #c (S1C Fig). As a result, ABE fermentation performance was also improved correspondingly (butanol: 11.63 g/L→13.91 g/L; acetone: 5.86 g/L→8.27 g/L) [38]. As a result, the novel fermentation strategy greatly stimulated lysine secretion by exogenous additions of either butyrate or acetate.

The proposed fermentation strategy also simultaneously enhanced *r*_GLC_^CA^ (Fig 3C) and *r*_NADH_^BtOH^ (Fig 3D). The large amount of lysine secretion and enhancement in *r*_NADH_^BtOH^ contributed to ABE fermentation performance enhancement co-operatively. More importantly, glucose consumption amount also largely increased from 51.3 g/L (case #a, control) to 69.3 g/L (case #d) with the proposed strategy. In this case, about 72% of the extra consumed glucose was utilized for ABE solvents production by *C*. *acetobutylicum*, as the metabolic activity of *S*. *cerevisiae* was severely inhibited, average contribution ratio of ethanol synthesis by *S*. *cerevisiae* reduced to 40–80% (*γ*≈0.4–0.8), and final ethanol concentration only accumulated at a lower level of 3.84 g/L. We must address that the severe ethanol synthesis (by *S*. *cerevisiae*) repression in this case (case #d) was somewhat different from that of “simulated” experiments implemented in the anaerobic bottles, which indicated that ethanol synthesis by *S*. *cerevisiae* were not further repressed so much under high butanol+butyrate (B&B) concentrations environments (10 g/L + 4 g/L, Fig 4A). This was because that, glucose rather than corn starch was used, co-existence of *C*. *acetobutylicum* and competition of the two strains on substrate utilizations in that case actually did not happen.

There would be a concern whether *S*. *cerevisiae* can really survive and fulfill the expected metabolic functions under the co-culturing environment. Therefore, we investigated *S*. *cerevisiae* cells viability and the theoretical glucose consumption by *S*. *cerevisiae* in the cases of co-culturing based ABE fermentations with/without exogenous butyrate addition, using CFU analysis method and the mathematical prediction model (Eq 2–Eq 10). As shown in Fig 6, the yeast cells could survive and glucose could be consumed by *S*. *cerevisiae* in both cases. However, viable yeast numbers were much less, *S*. *cerevisiae* metabolic activity and the glucose amount consumed by *S*. *cerevisiae* were much lower in the case of exogenously adding butyrate. As mentioned above, the co-culturing based ABE fermentations with/without exogenous butyrate addition could largely increase the total glucose consumption amount. Compared with the control, the extra glucose consumed amounts were 20 g/L (without butyrate addition) and 18 g/L (with butyrate addition), and the relevant extra glucose amounts consumed by the yeast were 14.3 g/L and 5.3 g/L respectively. In the case of co-culturing the two strains with butyrate addition, ratio of extra substrate consumed by *S*. *cerevisiae* decreased from 72% to 29%, glucose utilization ability/efficiency of *C*. *acetobutylicum* was enhanced significantly in an indirect manner.

**Fig 7:**
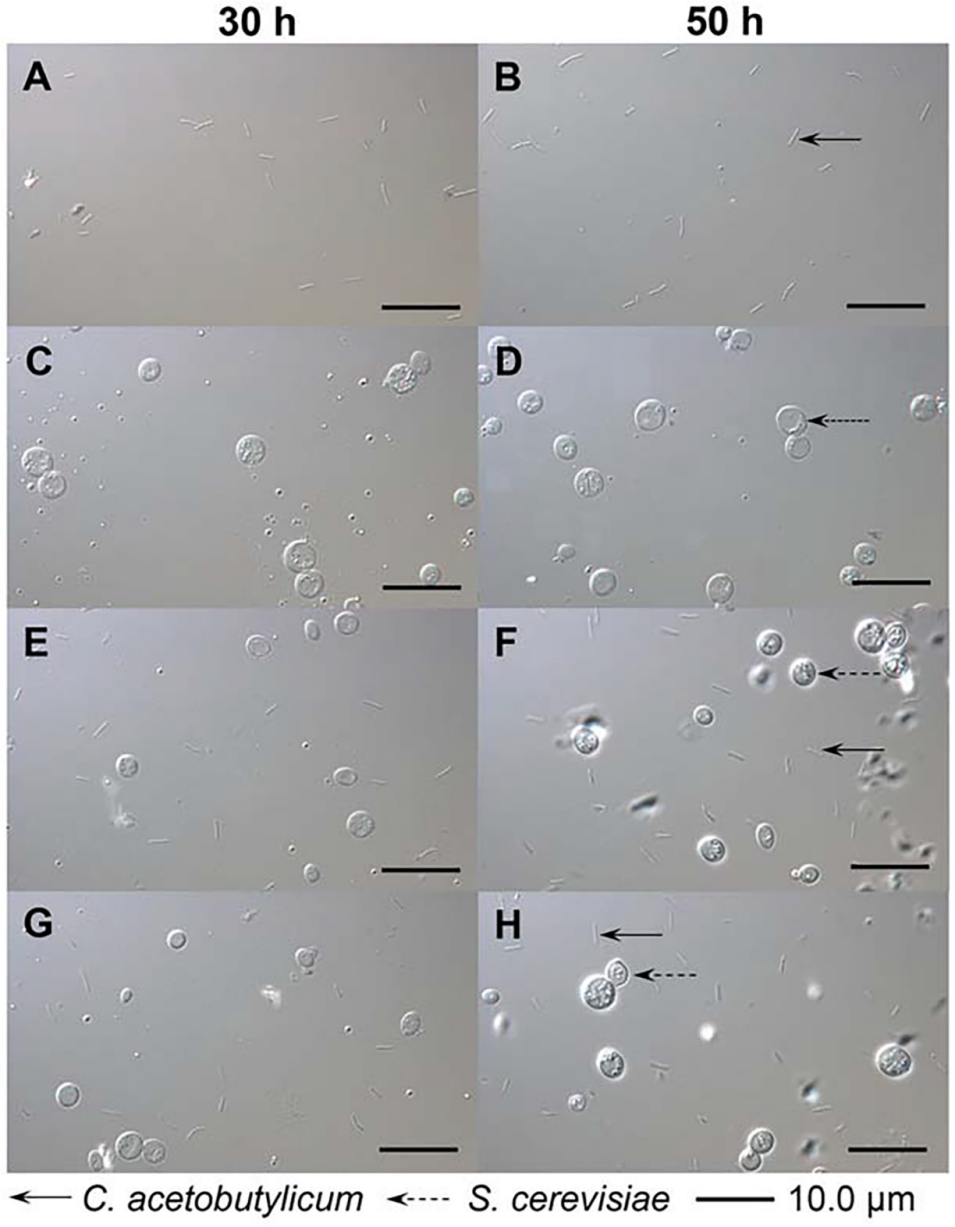
Morphological shapes of *C*. *acetobutylicum* and *S*. *cerevisiae* in pure cultivations and the proposed co-culturing system with/without exogenous butyrate addition. **(A)-(B)** The morphological shapes of *C*. *acetobutylicum* in pure *C*. *acetobutylicum* cultivation. **(C)-(D)** The morphological shapes of *S*. *cerevisiae* in pure *S*. *cerevisiae* cultivation (37°C). **(E)-(F)** The morphological shapes of *C*. *acetobutylicum* and *S*. *cerevisiae* in the co-culturing system without exogenous butyrate addition. **(G)-(H)** The morphological shapes of *C*. *acetobutylicum* and *S*. *cerevisiae* in the co-culturing system with exogenous butyrate addition.

Fig 7 showed the morphological shapes of *C*. *acetobutylicum* and *S*. *cerevisiae* (30 h and 50 h), in the pure cultivations of *C*. *acetobutylicum* and *S*. *cerevisiae*, as well as those in the *C*. *acetobutylicum*/*S*. *cerevisiae* co-culturing system with/without exogenous butyrate additions. The pictures clearly indicated that the two strains could co-exist without autolysis under the environments of 37°C and high concentrations of butanol or/and butyrate until the fermentation end (cases #c and #d). From Fig 7E–7H, it could be concluded that the amino acids secretions were originated from the viable yeast or/and *Clostridia* cells in the co-culturing system, rather than the release due to the yeast autolysis.

Finally, in 7 L fermentor, the proposed ABE fermentation strategy of co-culturing system integrated with exogenous butyrate addition increased butanol concentration from 11.63 g/L (control) to 15.74 g/L with an increment of 4.11 g/L. Therefore, there is a concern with regards to whether the titer increment was contributed by the added butyrate solely or by consuming glucose/butyrate co-operatively, since this is very important and closely related with the economics of the entire fermentation process. It was believed that, the enhanced butanol titer (4.11 g/L) originated from two parts: about 58% of the increment was from the extra consumed glucose (18.0 g/L), and another 42% was from the exogenously added butyrate (4.0 g/L-broth) directly. In our previous study [16], we investigated the theoretical conversion yield from exogenously added butyrate to butanol (*Y*_BtOH/BU_) based on graph theory and experimental data fitting, and this yield was determined as 48% (weight base, 0.48 g-butanol/g-butyrate). By considering this yield (0.48) and the residual butyrate concentration (0.39 g/L, Table 1), contribution of added butyrate to the butanol concentration increment would be about 42% ((4.0–0.39)×0.48/4.11 = 0.42). As for the rest 52% of consumed butyrate, we speculated that, they were assimilated in the *C*. *acetobutylicum* cells to maintain the fermentation under enhanced intercellular butyrate concentration environments to contribute another 58% butanol titer increment in an indirect way: 1) the higher intracellular butyrate concentration would induce excessive production of proton [H^+^], which is beneficial for butanol synthesis oriented NADH over-production. The exogenously added butyrate (molecule form) could penetrate into the cells, and then the dissociation equilibrium of CH_3_CH_2_CH_2_COOH ↔ CH_3_CH_2_CH_2_COO^−^+ H^+^ was formed intracellular [35]; 2) the higher intracellular butyrate concentration apparently stimulated the amino acid intracellular accumulation and secretion in either *C*. *acetobutylicum* or *S*. *cerevisiae*, some of the amino acids are favorable for *C*. *acetobutylicum* cells survival and butanol production, though we still do not understand the real mechanism of amino acids secretion under butyrate stress environment as experimental analysis of intracellular butyrate in *C*. *acetobutylicum* was very difficult in this study. On the other hand, based on the theoretical calculation of glucose consumed by *S*. *cerevisiae* (GLC^SC^) using Eq 10, it was estimated that 64.0 g/L glucose was utilized by *C*. *acetobutylicum* by deleting GLC^SC^ (5.3 g/L) from the total glucose consumed (69.3 g/L). In this case (case #d, Table 1) and in comparison with control, 12.7 g/L extra glucose was consumed by *C*. *acetobutylicum*. If the 12.7 g/L glucose was converted into butanol at the ABE yield on glucose of 39% and butanol ratio over total ABE of 60% [29], then 2.97 g/L butanol (12.7×39%×60% = 2.97 g/L) could be over-produced, which is roughly consistent with the estimation of 58% butanol titer enhancement originated from glucose (4.11×0.58 = 2.38 g/L, 2.38 g/L versus 2.97 g/L). As a result, though the apparent *Y*_BtOH_ and *Y*_ABE_ almost stayed at same levels as those of control, butanol yield on glucose by *C*. *acetobutylicum* (*Y'*_BtOH_) was increased from 0.227 to 0.246 (Table 1).

Besides the amino acids intracellular accumulation and extracellular secretions, the highest *r*_NADH_^BtOH^ and the extra glucose consumed by *C*. *acetobutylicum* achieved by the proposed fermentation strategy, the strategy might also affect the activities of the major intracellular enzymes regulating *C*. *acetobutylicum* metabolism. Among the enzymes, CoA-transferase (CoAT) encoded by *ctfAB* gene is most important, which could re-assimilate the organic acids (acetate and butyrate) formed, convert acetate and butyrate into acetyl-CoA and butyryl-CoA. As addressed many times, experimental analysis of the key enzymatic activities in *C*. *acetobutylicum* was very difficult in our case. However, we still measured the transcriptional levels of *ctfAB* in different batches by real-time fluorescence quantitative PCR analysis method. In solventogenesis phase, the normalized transcriptional level of *ctfAB* did not vary in different runs. The result indicated that, variations in butanol concentration/ratio were not due to variations in the transcriptional level. Table 1 summarized the comprehensive performance comparisons of ABE fermentations with different operation strategies.

### The application potential of the novel ABE fermentation strategy integrating *C*. *acetobutylicum*/*S*. *cerevisiae* co-culturing system with exogenous butyrate addition

Many investigators have reported the effect of exogenous butyrate addition and solventogenic *Clostridia* co-culturing mode on ABE fermentation performance. As shown in Table 2, exogenous butyrate addition (2.78–4.95 g/L) could enhance butanol concentration and butanol/acetone ratio by pure *Clostridia* cultivation. In the case of co-culturing *C*. *beijerinckii*/*C*. *tyrobutyricum* with glucose as the substrate, butanol concentration and ABE productivity enhanced by 33.3% and 173.3%, but butanol/acetone ratio decreased by 34.5%. With the proposed ABE fermentation strategy integrating *C*. *acetobutylicum*/*S*. *cerevisiae* co-culturing system with exogenous butyrate addition, butanol concentration, butanol/acetone ratio and total ABE productivity were enhanced 35%, 43% and 34%, respectively.

**Table 2:** Performance comparisons of ABE fermentations by butyrate supplementing or/and various co-culturing systems.

Culture mode	Strain(s)	Butyrate addition	Primary substrates	Butanol (g/L)	Acetone (g/L)	B/A ratio (g/g)	ABE (g/L)	ABE productivity (g/L/h)	Reference
Pure culture	*C*. *saccharoperbutylacetonicum* N1-4	None	Glucose	8.01	3.75	2.14	11.96	0.332	[40]
		4.95 g/L	Glucose	13.23	4.04	3.23	17.63	0.551	
Pure culture	*C*. *beijerinckii* NCIMB 8052	None	Glucose	7.80	2.70	2.89	10.96	0.126	[31]
		2.78 g/L	Glucose	10.2	3.00	3.40	13.01	0.271	
Pure culture	*C acetobutylicum* ATCC 824	None	Corn starch	11.11	5.72	1.94	18.06	0.361	[16]
		3.0 g/L	Corn starch	13.27	5.50	2.41	20.09	0.402	
Pure culture	*C*. *beijerinckii* ATCC 55025	None	Glucose	9.08	2.70	3.36	12.28	0.116	[20]
Co-culture^1^	*C*. *beijerinckii* ATCC 55025	None	Glucose	12.10	5.50	2.2	19.00	0.317	[20]
	+*C*. *tyrobutyricum* ATCC 25755								
Pure culture	*C*. *butylicum* TISTR 1032	None	Soluble starch	N/A	N/A	N/A	1.32	0.055	[19]
Co-culture^1^	*C*. *butylicum* TISTR 1032	None	Cassava starch	N/A	N/A	N/A	4.01	0.082	[19]
	+ *B*. *subtilis* WD 161								
Pure culture	*C acetobutylicum* ATCC 824	None	Corn starch	11.63	5.86	1.98	18.77	0.290	This study
Pure culture	*C acetobutylicum* ATCC 824	4.0 g/L	Corn starch	13.50	5.72	2.36	20.61	0.410	This study
Co-culture	*C acetobutylicum* ATCC 824	4.0 g/L	Corn starch	15.74	5.57	2.83	25.15	0.520	This study
	+ *S*. *cerevisiae*								

B/A ratio is butanol/acetone ratio; ABE refers to total solvents.

N/A: not available or supplied in literatures.

^1^: The two strains were inoculated into the fermentor simultaneously at the beginning of the fermentation.

There would be some arguments with regards to whether total ABE production and butanol ratio over total ABE solvents are really improved when using the proposed ABE fermentation strategy, as *S*. *cerevisiae* also produces ethanol which varies butanol ratio in turn. It was true that the apparent butanol ratio over total ABE solvents only slightly increased from 62% to 63% (compared with control, Table 1), which was even less than that of pure ABE fermentation with exogenous butyrate addition (from 62% to 66%, case #b). However, we must address that, with the novel fermentation strategy, butanol ratio actually increased from 62% to 70% by *C*. *acetobutylicum* metabolism, if ignoring the extra ethanol produced amount by *S*. *cerevisiae* (contribution ratio of 71%, Fig 3B). Nevertheless, the large increase in butanol concentration would improve the quality of the ABE solvents-mixture based diesel additives, as the butanol recovery ratio is the highest (96%) in the ABE purification system described by the literature [6], while the recovery ratio for ethanol is the lowest (50%). With the ABE mixtures achieved by the proposed strategy (butanol 15.74 g/L, acetone 5.57 g/L, ethanol: 3.84 g/L, Table 1), ABE ratio in the purified diesel additive could reach a desirable level of 74:17:9 (weight base). Many reports showed that, butanol ratio and total ABE concentration could be altered by the methods of metabolic engineering or gene knockout. Butanol ratio could increase from 70% to 80.05% by disrupting the acetoacetate decarboxylase gene (*adc*) in *C*. *acetobutylicum* EA 2018, but total ABE production decreased from 19.1 g/L to 9.3 g/L [8]. In another report, when the alcohol/aldehyde dehydrogenase gene (*aad*) was expressed from the phosphotransbutyrylase (*ptb*) promoter to enhance butanol synthesis and CoA-transferase (CoAT) was down-regulated to minimize acetone synthesis, high alcohol concentrations (butanol and ethanol: 13.19 g/L and 13.82 g/L), total ABE production (30.55 g/L) as well as butanol/acetone ratio (3.72) could be achieved, but butanol ratio reduced from 64.2% to 43.2% [39]. In addition, the unstable hereditary features of the engineered mutants limit their practical applications in fermentation industrial sectors. In this study, we attempted to increase overall ABE fermentation performance by traditional fermentation technique, using wild-type *C*. *acetobutylicum* and dominated raw material-corn starch. We believed that the novel fermentation strategy is industrially practical.

Butyrate is recognized as an excellent substrate for butanol production under the existence of glucose [13, 14]. However, the price of butyrate is equivalent to that of butanol, utilizing chemically synthesized butyrate as the co-substrate is not industrially feasible. In this study, we also conducted butyrate fermentation by *C*. *tyrobutyricum* in a 7 L anaerobic fermentor with glucose as the substrate, and then adding the butyrate containing supernatant into ABE fermentation broth, attempting to implement the proposed ABE fermentation process in a more economic way. The butyrate fermentation by *C*. *tyrobutyricum* ended at 60 h, and final butyrate concentration reached 28.5 g/L by consuming 75.2 g/L glucose. The yield of butyrate on glucose (*Y*_Bu/GLC_) was 0.38, which is much higher than butanol yield on glucose (*Y*_BtOH_, 0.23∼0.27). This provided the possibility for improving ABE fermentation economics. We used a vacuum flash-evaporation unit to distill water out at the top of the unit, and concentrated the fermentative butyrate supernatant from 28.5 g/L to 50.0 g/L at the bottom, then the concentrated butyrate supernatant subject to the pre-treatment was directly added into the co-culturing based ABE fermentation broth to replace the chemically synthesized butyrate. Concentrating the fermentative butyrate supernatant using the vacuum flash-evaporation unit and adding the concentrated supernatant into ABE fermentation broth were because that, we wished to avoid the big volume variations in ABE fermentation due to the dilution effect. As shown in Table 1, in this case (#e), butanol concentration also increased to 14.91 g/L in ABE fermentation by adding 300 mL of the concentrated supernatant which was equivalent to 4.0 g-butyrate/L-broth, with the ABE fermentation broth increment from 3.5 L to 3.8 L. Table 3 summarized a rough economical evaluation of proposed fermentation strategy by exogenously adding butyrate synthetic butyrate and the butyrate fermentative supernatant. From the table, it could be concluded that, the co-culturing system based ABE fermentations by adding either pure chemically synthesized butyrate or butyrate fermentative supernatant would have net gross profits, but the profits would reduce at certain extent in butanol and total ABE productions when considering extra starch consumptions induced by the butyrate additions as compared with those of the standard ABE fermentation (control). However, we believed that the gross profits decrease in those cases could be compensated by the high ABE productivity, high butanol ratio, and the reduced purification cost for distilling fermentation broth with higher butanol concentrations. Finally, we must address that the proposed ABE fermentation strategy by co-culturing *C*. *acetobutylicum*/*S*. *cerevisiae* integrating with exogenous butyrate stress is only effective for corn-based ABE fermentations by *C*. *acetobutylicum*, which on the other hand, still dominate the current industrial bio-butanol production in the world.

**Table 3:** Economical evaluation of the proposed fermentation strategy using synthetic butyrate and butyrate fermentative supernatant.

**Butanol (t/1,000m** ^**3**^ **-$)**		**Butanol**		**Butyrate**	**Corn-starch** *	**Glucose**		**Gross profit**
Considering butyrate addition alone								
Control, case #a		11.63/20,900		0/0	47/14,100	0/0		6,800
Case #d		15.74/28,300		4/7,000	47/14,100	0/0		7,200
Case #e		14.91/26,850		0/0	47/14,100	10.5**/4,200		8,550
Comprehensive case considering extra starch consumption by butyrate addition in ABE fermentation								
Control, case #a		11.63/20,900		0/0	47/14,100	0/0		6,800
Case #d		15.74/28,300		4/7,000	63/18,900	0/0		2,400
Case #e		14.91/26,850		0/0	55.5/16,650	10.5**/4,200		6,000
**Total ABE (t/1,000m** ^**3**^ **-$)**		**ABE solvents**		**Butyrate**	**Corn-starch** *	**Glucose**		**Gross profit**
Considering butyrate addition alone								
Control, case #a		18.77/31,590		0/0	47/14,100	0/0		17,490
Case #d		25.15/41,000		4/7,000	47/14,100	0/0		19,900
Case #e		23.05/38,300		0/0	47/14,100	10.5**/4,200		20,000
Comprehensive case considering extra starch consumption by butyrate addition in ABE fermentation								
Control, case #a		18.77/31,590		0/0	47/14,100	0/0		17,490
Case #d		25.15/41,000		4/7,000	63/18,900	0/0		15,100
Case #e		23.05/38,300		0/0	55.5/16,650	10.5**/4,200		17,450
**Unit price**	**Corn-starch**		**Glucose**	**Butyrate**	**Butanol**		**Acetone**	**Ethanol**
**($/ton)**	300		400	1,750	1,800		1,600	1,000

* Corn-starch consumption was calculated by 0.9×(glucose consumed amount) in ABE fermentation.

** Butyrate yield on glucose in butyrate fermentation was 0.38. The glucose consumed amount in butyrate fermentation was 10.5 g/L glucose (1/0.38×4 g-glucose/L).

## Supporting Information

S1 Fig Amino acids secretion patterns with different operation strategies in 7 L fermentor when exogenously adding acetate. **(A)** ABE fermentation by *C*. *acetobutylicum* (control, case #a). **(B)** ABE fermentation by *C*. *acetobutylicum* with exogenous acetate addition (4.0 g/L-broth). **(C)** ABE fermentation by co-culturing *C*. *acetobutylicum*/*S*. *cerevisiae* (0.20 g-DCW/L-broth) in coupling with exogenous acetate addition (4.0 g/L-broth). **(D)** ABE fermentation by co-culturing *C*. *acetobutylicum*/*S*. *cerevisiae* (0.20 g-DCW/L-broth) in coupling with exogenously adding acetate fermentation supernatant (acetic acid fermentation broth, acetate concentration 75 g/L, pure acetate adding amount of 8.0 g/L-broth).Black: lysine; white: methionine; horizontal shadow: phenylalanine; slashed shadow: tyrosine.
(EPS)

## Abbrevations

ACE: acetone
BtOH: butanol
EtOH: ethanol or apparent ethanol concentration
EtOH^CA^: concentration of ethanol synthesized by *C*. *acetobutylicum* (g/L)
GLC: glucose
GLC^SC^: extra glucose consumed amount by *S*. *cerevisiae* (g/L)
MW_EtOH_: molecular weight of ethanol
MW_GLC_: molecular weight of glucose
*r*_ACE_: acetone synthesis rate (mol/L/h)
*r*_CO2_: CO_2_ evolution rate (mol/L/h)
*r*_CO2_^CA^: CO_2_ evolution rate by *C*. *acetobutylicum* (mol/L/h)
*r*_CO2_^SC^: CO_2_ evolution rate by *S*. *cerevisiae* (mol/L/h)
*r*_EtOH_: total (apparent) ethanol production rate (mol/L/h)
*r*_EtOH_^CA^: ethanol production rate by *C*. *acetobutylicum* (mol/L/h)
*r*_EtOH_^SC^: ethanol production rate by *S*. *cerevisiae* (mol/L/h)
*r*_GLC_: total (apparent) glucose utilization rate (mol/L/h)
*r*_GLC_^CA^: glucose utilization rate in *C*. *acetobutylicum* (mol/L/h)
*r*_GLC_^SC^: glucose utilization rate in *S*. *cerevisiae* (mol/L/h)
*r*_H2_: H_2_ evolution rate (mol/L/h)
*r*_NADH_^BtOH^: butanol synthesis oriented NADH utilization rate in *C*. *acetobutylicum* (mol/L/h)
γ: contribution ratio (mol base) of ethanol production by *S*. *cerevisiae* over total ethanol production, or *r*_EtOH_^SC/^ *r*_EtOH_

## References

[1] Lee SY, Park JH, Jang SH, Nielsen LK, Kim J, Jung KS. Fermentative butanol production by *Clostridia* . Biotechnol Bioeng. 2008; 101(2):209–28. 10.1002/bit.22003 18727018

[2] Dürre P. Biobutanol: an attractive biofuel. Biotechnol J. 2007; 2(12):1525–34. 17924389 10.1002/biot.200700168

[3] Wu H, Nithyanandan K, Zhou N, Lee TH, Lee C-F, Zhang C. Impacts of acetone on the spray combustion of acetone-butanol-ethanol (ABE)-diesel blends under low ambient temperature. Fuel. 2015; 142:109–16.

[4] Jang Y-S, Lee JY, Lee J, Park JH, Im JA, Eom M-H, et al Enhanced butanol production obtained by reinforcing the direct butanol-forming route in *Clostridium acetobutylicum* . MBio. 2012; 3(5):e00314–12. 10.1128/mBio.00314-12 23093384 PMC3482502

[5] Lee S-H, Kwon M-A, Shin Y-A, Kim KH. Butanol production by metabolically engineered *Clostridium acetobutylicum* with in-situ butanol removal. New Biotechnol. 2014; (31):S102.

[6] Liu J, Fan L, Seib P, Friedler F, Bertok B. Downstream process synthesis for biochemical production of butanol, ethanol, and acetone from grains: generation of optimal and near-optimal flowsheets with conventional operating units. Biotechnol Prog. 2004; 20(5):1518–27. 15458338 10.1021/bp049845v

[7] Bahl H, Gottwald M, Kuhn A, Rale V, Andersch W, Gottschalk G. Nutritional factors affecting the ratio of solvents produced by *Clostridium acetobutylicum* . Appl Environ Microbiol. 1986; 52(1):169–72. 16347104 10.1128/aem.52.1.169-172.1986 PMC203431

[8] Jiang Y, Xu C, Dong F, Yang Y, Jiang W, Yang S. Disruption of the acetoacetate decarboxylase gene in solvent-producing *Clostridium acetobutylicum* increases the butanol ratio. Metab Eng. 2009; 11(4):284–91. 19560551 10.1016/j.ymben.2009.06.002

[9] Wang S, Zhu Y, Zhang Y, Li Y. Controlling the oxidoreduction potential of the culture of *Clostridium acetobutylicum* leads to an earlier initiation of solventogenesis, thus increasing solvent productivity. Appl Microbiol Biotechnol. 2012; 93(3):1021–30. 10.1007/s00253-011-3570-2 21935591

[10] Girbal L, Vasconcelos I, Saint-Amans S, Soucaille P. How neutral red modified carbon and electron flow in *Clostridium acetobutylicum* grown in chemostat culture at neutral pH. FEMS Microbiol Rev. 1995; 16(2–3):151–62.

[11] Peguin S, Goma G, Delorme P, Soucaille P. Metabolic flexibility of *Clostridium acetobutylicum* in response to methyl viologen addition. Appl Microbiol Biotechnol. 1994; 42(4):611–6.

[12] Du Y, Jiang W, Yu M, Tang I, Yang ST. Metabolic process engineering of *Clostridium tyrobutyricum* Δ*ack-adhE2* for enhanced n-butanol production from glucose: effects of methyl viologen on NADH availability, flux distribution, and fermentation kinetics. Biotechnol Bioeng. 2014; 112(4):705–15. 10.1002/bit.25489 25363722

[13] Tashiro Y, Takeda K, Kobayashi G, Sonomoto K, Ishizaki A, Yoshino S. High butanol production by *Clostridium saccharoperbutylacetonicum* N1-4 in fed-batch culture with pH-Stat continuous butyric acid and glucose feeding method. J Biosci Bioeng. 2004; 98(4):263–8. 16233703 10.1016/S1389-1723(04)00279-8

[14] Tashiro Y, Shinto H, Hayashi M, Baba S-i, Kobayashi G, Sonomoto K. Novel high-efficient butanol production from butyrate by non-growing *Clostridium saccharoperbutylacetonicum* N1-4 (ATCC 13564) with methyl viologen. J Biosci Bioeng. 2007; 104(3):238–40. 17964492 10.1263/jbb.104.238

[15] Li Z, Shi Z, Li X, Li L, Zheng J, Wang Z. Evaluation of high butanol/acetone ratios in ABE fermentations with cassava by graph theory and NADH regeneration analysis. Biotechnol Bioprocess Eng. 2013; 18(4):759–69.

[16] Li X, Shi Z, Li Z. Increasing butanol/acetone ratio and solvent productivity in ABE fermentation by consecutively feeding butyrate to weaken metabolic strength of butyrate loop. Bioprocess Biosyst Eng. 2014; 37:1609–16. 10.1007/s00449-014-1133-5 24500620

[17] Chang J-J, Chou C-H, Ho C-Y, Chen W-E, Lay J-J, Huang C-C. Syntrophic co-culture of aerobic *Bacillus* and anaerobic *Clostridium* for bio-fuels and bio-hydrogen production. Int J Hydrogen Energy. 2008; 33(19):5137–46.

[18] Bader J, Mast-Gerlach E, Popović M, Bajpai R, Stahl U. Relevance of microbial coculture fermentations in biotechnology. J Appl Microbiol. 2010; 109(2):371–87. 10.1111/j.1365-2672.2009.04659.x 20070440

[19] Tran HTM, Cheirsilp B, Hodgson B, Umsakul K. Potential use of *Bacillus subtilis* in a co-culture with *Clostridium butylicum* for acetone-butanol-ethanol production from cassava starch. Biochem Eng J. 2010; 48(2):260–7.

[20] Li L, Ai H, Zhang S, Li S, Liang Z, Wu Z-Q, et al Enhanced butanol production by coculture of *Clostridium beijerinckii* and *Clostridium tyrobutyricum* . Bioresour Technol. 2013; 143:397–404. 10.1016/j.biortech.2013.06.023 23819976

[21] Ashe MP, Slaven JW, Long SKD, Ibrahimo S, Sachs AB. A novel eIF2B-dependent mechanism of translational control in yeast as a response to fusel alcohols. EMBO J. 2001; 20(22):6464–74. 11707417 10.1093/emboj/20.22.6464 PMC125737

[22] Sun Z, Wang S, Wu Y. The production of acetone-butanol with immobilized cells of *Clostridium acetobutylicum* . Chin J Ind Microbiol. 1987; 17(6):18–22.

[23] Zhang L, Yang Y, Shi Z. Performance optimization of property-improved biodiesel manufacturing process coupled with butanol extractive fermentation. Chin J Biotechnol. 2008; 24(11):1943–8. 19256343

[24] Luo HZ, Zhang JS, Yuan GQ, Zhao YL, Liu H, He ZN, et al Performance improvement of cephalosporin C fermentation by *Acremonium chrysogenum* with DO-Stat based strategy of co-feeding soybean oil and glucose. Process Biochem. 2013; 48(12):1822–30.

[25] Li X, Li Z, Zheng J, Shi Z, Li L. Yeast extract promotes phase shift of bio-butanol fermentation by *Clostridium acetobutylicum* ATCC824 using cassava as substrate. Bioresour Technol. 2012; 125:43–51. 10.1016/j.biortech.2012.08.056 23023236

[26] Fontaine L, Meynial-Salles I, Girbal L, Yang X, Croux C, Soucaille P. Molecular characterization and transcriptional analysis of *adhE2*, the gene encoding the NADH-dependent aldehyde/alcohol dehydrogenase responsible for butanol production in alcohologenic cultures of *Clostridium acetobutylicum* ATCC 824. J Bacteriol. 2002; 184(3):821–30. 11790753 10.1128/JB.184.3.821-830.2002 PMC139506

[27] Girbal L, Soucaille P. Regulation of solvent production in *Clostridium acetobutylicum* . Trends Biotechnol. 1998; 16(1):11–6.

[28] Yu M, Zhang Y, Tang I-C, Yang S-T. Metabolic engineering of *Clostridium tyrobutyricum* for n-butanol production. Metab Eng. 2011; 13(4):373–82. 10.1016/j.ymben.2011.04.002 21530675

[29] The Japan Institute of Energy. Biomass Handbook. Tokyo: Ohmsha Publishing Co., Ltd.; 2002. 422 p.

[30] Baba S-i, Tashiro Y, Shinto H, Sonomoto K. Development of high-speed and highly efficient butanol production systems from butyric acid with high density of living cells of *Clostridium saccharoperbutylacetonicum* . J Biotechnol. 2012; 157(4):605–12. 10.1016/j.jbiotec.2011.06.004 21683741

[31] Wang Y, Li X, Blaschek HP. Effects of supplementary butyrate on butanol production and the metabolic switch in *Clostridium beijerinckii* NCIMB 8052: genome-wide transcriptional analysis with RNA-Seq. Biotechnol Biofuels. 2013; 6(1):138 10.1186/1754-6834-6-138 24229082 PMC3849199

[32] Masion E, Amine J, Marczak R. Influence of amino acid supplements on the metabolism of *Clostridium acetobutylicum* . FEMS Microbiol Lett. 1987; 43(3):269–74.

[33] Heluane H, Evans MR, Dagher SF, Bruno-Bárcena JM. Meta-analysis and functional validation of nutritional requirements of solventogenic *Clostridia* growing under butanol stress conditions and coutilization of D-glucose and D-xylose. Appl Environ Microbiol. 2011; 77(13):4473–85. 10.1128/AEM.00116-11 21602379 PMC3127714

[34] Alsaker KV, Paredes C, Papoutsakis ET. Metabolite stress and tolerance in the production of biofuels and chemicals: gene-expression-based systems analysis of butanol, butyrate, and acetate stresses in the anaerobe *Clostridium acetobutylicum* . Biotechnol Bioeng. 2010; 105(6):1131–47. 10.1002/bit.22628 19998280

[35] Ezeji T, Milne C, Price ND, Blaschek HP. Achievements and perspectives to overcome the poor solvent resistance in acetone and butanol-producing microorganisms. Appl Microbiol Biotechnol. 2010; 85(6):1697–712. 10.1007/s00253-009-2390-0 20033401

[36] Knoshaug EP, Zhang M. Butanol tolerance in a selection of microorganisms. Appl Biochem Biotechnol. 2009; 153(1–2):13–20. 19089652 10.1007/s12010-008-8460-4

[37] Fischer CR, Klein-Marcuschamer D, Stephanopoulos G. Selection and optimization of microbial hosts for biofuels production. Metab Eng. 2008; 10(6):295–304. 10.1016/j.ymben.2008.06.009 18655844

[38] Li Z. Improvement of butanol concentration and butanol/acetone ratio in Acetone-Butanol-Ethanol fermentation by metabolic network based graph theory [Ph.D. Thesis]. Wuxi: Jiangnan University; 2014.

[39] Sillers R, Al-Hinai MA, Papoutsakis ET. Aldehyde-alcohol dehydrogenase and/or thiolase overexpression coupled with CoA transferase downregulation lead to higher alcohol titers and selectivity in *Clostridium acetobutylicum* fermentations. Biotechnol Bioeng. 2009; 102(1):38–49. 10.1002/bit.22058 18726959

[40] Ventura J-R, Jahng D. Improvement of butanol fermentation by supplementation of butyric acid produced from a brown alga. Biotechnol Bioprocess Eng. 2013; 18(6):1142–50.

